# Supplementary material for: In Vivo Imaging Reveals Extracellular Vesicle-Mediated Phenocopying of Metastatic Behavior
Source: Cell. 2015 May 21;161(5):1046–57. doi: 10.1016/j.cell.2015.04.042 (PMC4448148; doi:10.1016/j.cell.2015.04.042)
Supplement: Document S1. Supplemental Experimental Procedures [file mmc1.pdf]

Cell, Volume *161*
Supplemental Information

In Vivo Imaging Reveals Extracellular Vesicle-Mediated Phenocopying of Metastatic Behavior

Anoek Zomer, Carrie Maynard, Frederik Johannes Verweij, Alwin Kamermans, Ronny Schäfer, Evelyne Beerling, Raymond Michel Schiffelers, Elzo de Wit, Jordi Berenguer, Saskia Inge Johanna Ellenbroek, Thomas Wurdinger, Dirk Michiel Pegtel, and Jacco van Rheenen

Supplemental Experimental Procedures

Cell culture and generation of stable cell lines

MDA-MB-231 cells (a kind gift from Dr. Jeffrey Segall) and B16 cells were cultured in Dulbecco's Modified Eagle's Medium + GlutaMAX (DMEM; GIBCO, Invitrogen Life Technologies, Paisley, UK) supplemented with 10% (v/v) fetal bovine serum (Sigma, St. Louis, MO, USA), 100 µg/ml streptomycin, and 100 U/ml penicillin (Invitrogen Life Technologies, Paisley, UK)(complete culture medium). MCF-7 and T47D cells were cultured in DMEM/F12 + GlutaMAX (GIBCO, Invitrogen Life Technologies, Paisley, UK) supplemented with 10% (v/v) fetal bovine serum, 100 µg/ml streptomycin, and 100 U/ml penicillin. Cells were kept at 37°C in a humidified atmosphere containing 5% CO_2. The Cre(ER^{T2}) and reporter$^+$ cell lines were generated using a standard lentiviral transduction or Lipofectamine 2000^{tm} (Invitrogen Life Technologies, Paisley, UK) protocol. Afterwards, cells were sorted by flow cytometry, cultured as a polyclonal population and kept under selection using puromycin (Gibco Life Technologies, Paisley, UK) or Zeocin (Invitrogen Life Technologies, Paisley, UK).

DNA constructs and stable cell lines

The cDNAs of $CreER^{T2}$, CFP and floxed-DsRed-floxed-eGFP were cloned into a pLV.CMV construct. The cDNA of Cre was cloned into a pcDNA3.1 construct. Multiple rounds of flow cytometry were performed to obtain floxed-DsRed-floxed-eGFP-expressing cell lines that were negative for eGFP. After prolonged culturing of the cells, flow cytometry was repeated.

Tamoxifen treatment of $CreER^{T2}$ cells and tumors

To activate the tamoxifen-inducible Cre variant ($CreER^{T2}$), 5 mg tamoxifen (Sigma, St. Louis, MO, USA) was injected intraperitoneally twice a week in mice bearing tumors of ≥ 5 mm^3 (Figure 4E (MDA-MB-231 and PyMT tumors), Figure S2, and Figure S6), or for *in vitro* culture

experiments cells were treated with 4-OH tamoxifen (3 μM; Sigma, St. Louis, MO, USA; Figure 3A-C and Figure S6A). For the experiments involving T47D and MCF-7, tamoxifen could not be used since these lines require estrogen to grow *in vivo*. For experiments involving these lines, and other experiments that required a direct comparison between various lines, the "normal" Cre variant was used that does not require tamoxifen induction for activity (Figure 3D-E, Figure 4B-D, Figure 4E (T47D and B16 tumors), Figure 5, Figure 6, Figure 7, Figure S3, Figure S4 and Figure S5).

Co-cultures and transwell experiments

For all co-culture and transwell experiments, complete DMEM culture medium was used. MDA-MB-231 Cre$^+$ cells were co-cultured with MCF-7, T47D or MDA-MB-231 reporter$^+$ cells for one week prior to imaging in a culture dish. All experiments were done in parallel. For the transwell experiments, transwells with a pore-size of 400 nm (Greiner Bio-one, Frickenhause, Germany) were placed in a culture dish with reporter$^+$ cells for one week prior to imaging. The cells in the culture dish were imaged with a Leica AF7000 microscope (Mannheim, Germany) using a 10x objective. During imaging, cells were kept at 37°C in a humidified atmosphere containing 5% CO_2. A 430/24 nm excitation filter and a 470/40 nm emission filter was used for CFP, a 470/40 nm excitation filter and a 520/40 nm emission filter was used for eGFP, and a 572/35 nm excitation filter and a 640/50 nm emission filter was used for DsRed. Every 30 minutes CFP, eGFP and DsRed images were acquired for 15 hours, and the images were analyzed in ImageJ (ImageJ, U. S. NIH, Bethesda, Maryland, USA, http://imagej.nih.gov/ij/, 1997-2011). For 2D migration experiments cells were tracked manually with an ImageJ plugin ("Manual Tracking" Rasband, W.S., ImageJ, U. S. NIH, Bethesda, Maryland, USA, http://imagej.nih.gov/ij/, 1997-2011.). At the beginning of each movie, a random DsRed$^+$ cell close to an eGFP$^+$ cell was selected. The *XY* position was determined over time and the migration speed was calculated by Excel (Microsoft).

Mice

MMTV-PyMT (pure FVB background), ACTB-Cre and R26-LoxP-STOP-LoxP(LSL)-tdTomato (both pure black6 (B6) background) mice were purchased from Jackson Laboratory, Sacramento, CA, USA. R26-CreER^{T2} and R26-Confetti mice were kind gifts from respectively the lab of Dr. Jacqueline Deschamps and the lab of Dr. Hans Clevers. Crossings to obtain experimental MMTV-PyMT;R26-CreER^{T2} or MMTV-PyMT;R26-Confetti mice were performed on a mixed genetic background. Immune-competent mice were housed under standard laboratory conditions and NOD *scid* gamma (NSG) mice (own crossing) were housed under IVC conditions. Mice received food and water ad libitum. All experiments were carried out in accordance with the guidelines of the Animal Welfare Committee of the Royal Netherlands Academy of Arts and Sciences, The Netherlands.

Injection of tumor cells

One week before injection of Cre^{+} and reporter^{+} tumor cells that included T47D and/or MCF-7 tumor cells, mice were ovariectomized and implanted with an estrogen pellet (0.72 mg/pellet, 60-day release, Innovative Research of America, Sarasota, FL, USA) using a precision trochar, as per the manufacturer's instructions. For injections that did not include T47D and/or MCF-7 tumor cells, mice were not overiectomized. For tumor development, 5 x 10^5 MDA-MB-231 cells, 1 x 10^6 T47D/MCF-7 cells, 1 x 10^5 B16 cells or mixtures of 2.5 x 10^5 MDA-MB-231 cells and 5 x 10^5 T47D/MCF-7 cells were injected in the fourth and/or ninth mammary fat pad of female NSG mice (for the MDA-MB-231, T47D and MCF-7 cell lines) or B6 mice (for the B16 melanoma cell lines) of 8-20 weeks old. Prior to injection, MDA-MB-231 or B16 cells were resuspended in sterile PBS and T47D or MCF-7 cells were resuspended in sterile PBS with 50% growth factor-reduced matrigel (BD Biosciences, Franklin Lakes, NJ, USA).

Transplantation of tumor pieces

MMTV-PyMT;R26-CreER^{T2} or MMTV-PyMT;R26-Confetti tumors were dissected aseptically from mice, cut into 2-3 mm^3 tumor pieces and stored in liquid N_2 in 80% FBS/20% DMSO until transplantation. Tumor pieces were implanted in the fourth and/or ninth mammary fat pad of NSG mice through a small skin incision. The incision was closed with a sterile 4-0 prolene suture (Ethicon, San Angelo, Texas, USA). 5 mg tamoxifen (Sigma, St. Louis, MO, USA) was injected intraperitoneally twice a week in mice bearing tumors ≥ 5 mm^3. Mice bearing tumors of ~10 mm^3 were sacrificed and tumors were removed.

Tumor and tissue processing

Tumors and tissues were removed from the mice at the end of the experiment and fixed in periodate-lysine-paraformaldehyde (PLP) buffer (2.5 ml 4% PFA + 0.0212 g $NaIO_4$ + 3.75 ml L-Lysine + 3.75 ml P-buffer (pH 7.4)) O/N at 4°C. The following day, the fixed tumors and tissues were washed twice with P-buffer and placed for at least 6 hours in 30% sucrose at 4°C. The tumors and tissues were then embedded in tissue freezing medium (Leica Microsystems, Nussloch, Germany) and stored at -80°C before cryosectioning.

Immunostainings and confocal microscopy of tissue sections

Tumor or tissue cryosections (15 µm thick when used for subsequent immunostainings, 100-150 µm thick for all other experiments) were rehydrated for 10 min in Tris 0.1 M pH 7.4, when indicated used for immunostainings and embedded in Vectashield mounting medium (hard set; Vector Labs, Burlington, Ontario, Canada). Immunostainings were performed using the following primary antibodies: rat anti-mouse CD45 biotin (eBioscience, San Diego, CA, USA; dilution 1:500), rat anti-mouse Ly-6G (Gr-1) (eBioscience, San Diego, CA, USA; dilution 1:100) and rat anti-mouse F4/80 biotin (Life Technologies, Carlsbad, CA, USA; dilution 1:400). Streptavidin-647 (Life Technologies, Carlsbad, CA, USA; dilution 1:500) and anti-rat Alexa-647 (Invitrogen Life Technologies, Paisley, UK; dilution 1:500) were used as secondary antibodies. When

indicated, cryosections were counterstained with 0.1 µg/ml DAPI (Invitrogen Life Technologies, Paisley, UK) to visualize the nuclei. Images were acquired using a Leica SP5 or SP8 AOBS confocal microscope (Mannheim, Germany) equipped with 10x NA 0.3 and 20x NA 0.7 dry objectives. DAPI and CFP were excited with a UV 405 nm laser, and emission was collected at 415-455 nm for DAPI and 455-495 nm for CFP. eGFP and YFP were excited with an argon ion laser (at 488 nm and 514 nm respectively) and emission was collected at 490-515 nm for eGFP and 520-560 nm for YFP. DsRed and tdTomato were excited with a 561 nm laser and emission was collected at 570-620 nm. Alexa-647 was excited with a 639 nm laser and emission was collected at 620-675 nm. At least 45 frozen sections per mouse were analyzed.

Isolation and injection of EVs

To isolate EVs from cells grown *in vitro*, cells were cultured in 200 ml medium supplemented with EV-free FBS (FBS centrifuged for 1 h at 70,000 *g*) for 48-72 h. To isolate EVs from primary PyMT tumors, tissue was dissociated using Liberase (Roche, Basel, Schweiz) without mechanical dissociation (to prevent breakage of cells): tumor pieces were incubated for 30 min at 37°C in Liberase (5 Wünsch units/ml)/DNaseI (25 µg/ml; Roche, Basel, Schweiz). Cells and cellular debris were removed from the cell culture or primary tumor supernatant by differential centrifugation: 2 x 500 *g* for 10 min, 2 x 2,000 *g* for 15 min, and 2 x 16,500 *g* for 20 min. EVs were pelleted by high-speed centrifugation at 70,000 *g* for 1 h, washed with PBS, and pelleted again by high-speed centrifugation at 70,000 *g* for 1 h. All high-speed centrifugation steps were performed at 4°C using a Beckman Avanti J-30I with a JS-24.38 rotor (Beckman Coulter, Fullerton, CA, USA). Finally, EVs were resuspended in 200 µl PBS, analyzed by electron microscopy and NanoSight to determine purity and size distribution of the EVs and/or used for injection experiments. Before the injection of EVs in reporter^{+} tumors in mice, EVs were treated with 20 µg/ml proteaseK (protK; Promega, Madison, WI, USA) followed by heat inactivation at 60°C for 10 min and 10 µg/ml RNaseA (Roche, Basel, Schweiz) followed by heat inactivation at

60°C for 10 min. To exclude that reporter^{+} tumor cells report random uptake of free non-vesicular-Cre, we intratumorally injected 110 ng recombinant Cre protein (NEB, Ipswich, MA, USA) or 10% lysate of a Cre^{+} MDA-MB-231 tumor (the tumor was dissociated, cells were pelleted and lysed in a 1% Triton lysis buffer without SDS to minimize denaturation of proteins).

PKH67-labeling of EVs

EVs were labelled with PKH67 green fluorescent membrane linker-dye (Sigma, St. Louis, MO, USA) according to the manufacturer's protocol. Labelled EVs were washed in 30 ml medium supplemented with EV-free FBS and collected by high-speed centrifugation as described above. PKH67-labelled EVs were added to MDA-MB-231 cells seeded in glass bottom culture dishes (Greiner Bio-one, Frickenhausen Germany). Internalization of EVs was assessed by confocal microscopy at a Leica SP5 AOBS confocal microscope (Mannheim, Germany) equipped with a dry 20x N.A. 0.7 objective.

Western blot

Cells or EVs were lysed in 1% SDS buffer and equal amounts of protein were loaded onto an SDS/PAGE gel. Antibodies against the following proteins/epitopes were used with the sources and dilution ratios indicated in parentheses: Cre (Millipore, Darmstadt, Germany; 1:1000, non-reducing conditions), Hsp70 (Santa Cruz Biotechnology, Santa Cruz, CA, USA; 1:200), CD63 (BD Pharmingen, San Jose, CA, USA; 1:200, non-reducing conditions) and Rab27A (Santa Cruz Biotechnology, Santa Cruz, CA, USA; 1:250).

Reverse transcriptase PCR

RNA was isolated using Trizol reagent (Invitrogen Life Technologies, Paisley, UK) according to the manufacturer's protocol and stored at -80°C. The amount and purity of isolated RNA was analyzed by the Nanodrop spectrophotometer (Wilmington, DE, USA). cDNA was prepared

using the Avian Myeloblastosis Virus Reverse Transcriptase kit (Promega, Madison, WI, USA) according to the manufacturer's instructions. cDNA was amplified using a PCR with Cre-specific primers (forward primer: 5' GCCTGCATTACCGGTCGATGC 3'; reverse primer: 5' GTGGCAGATGGC GCGGCAACA 3'). Thermal cycle conditions used for all reactions were as follows: 5 min at 95°C, followed by 40 cycles consisting of denaturation for 30 sec at 95°C, annealing for 30 sec at 58°C, and extension for 1 min at 72°C. PCR reactions were concluded with incubation for 10 min at 72°C to complete the extension of all synthesized products. PCR products were then visualized on a 1.25% TAE agarose gel.

Electron microscopy and NanoSight particle analysis

EV size distributions were analyzed using a NanoSight LM10 system (NanoSight, Amesbury, UK). The system calculates size by tracking the Brownian motion of individual nanoparticles detected by scattered laser light as a function of dispersing medium viscosity and temperature. Samples were diluted until individual nanoparticles could be tracked. Samples were measured five times and analyzed using Nanoparticle Tracking Analysis software (NanoSight).

For electron microscopy, EVs were fixed with 2% paraformaldehyde/0.2% glutaraldehyde solution, coated on a copper grid and post-stained with uranyl oxale acetate and methylcellulose uranyl acetate. Samples were viewed on a FEI Tecnai T12 transmission electron microscope (FEI, Hillsboro, Oregon, USA), and images were digitally taken using a CCD camera and FEI version 3.2 TEM Imaging and Analysis Software.
