# Supplementary figures and images for: In Vivo Imaging Reveals Extracellular Vesicle-Mediated Phenocopying of Metastatic Behavior
Source: Cell. 2015 May 21;161(5):1046–57. doi: 10.1016/j.cell.2015.04.042 (PMC4448148; doi:10.1016/j.cell.2015.04.042)

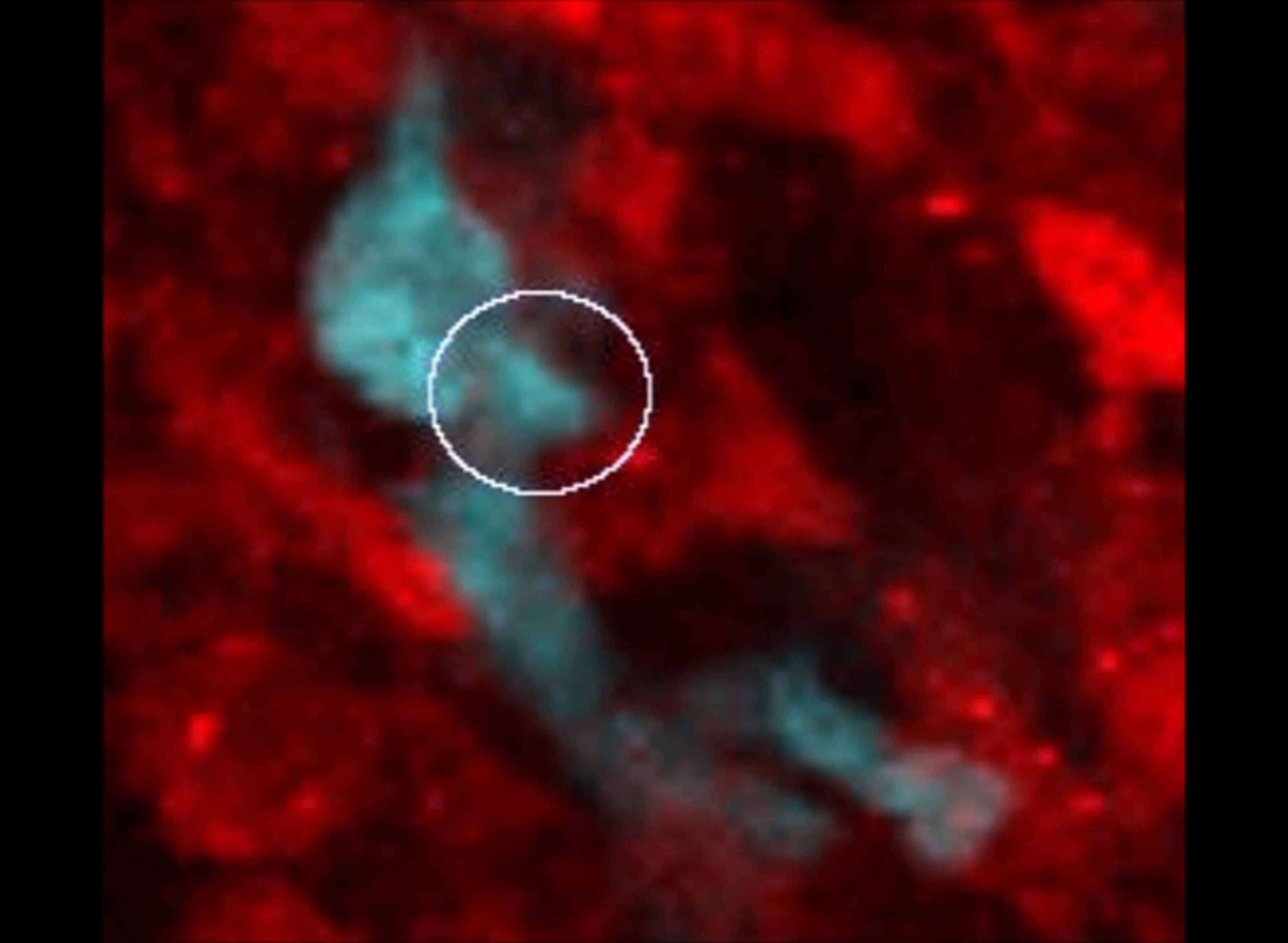

Supplement: Movie S1. Intravital Imaging Movie Example 1 Showing the Release of an Extracellular Vesicle, Related to Figure 1 — Shown is an intravital movie that was acquired of a MDA-MB-231 tumor consisting of cells expressing different fluorescent proteins. All frames in the movie represent maximum projections of 5 z planes with a total z volume of 25 μm. The cell that releases an extracellular vesicle is indicated in cyan, and the released EV is marked with a circle. [file mmc4.jpg]

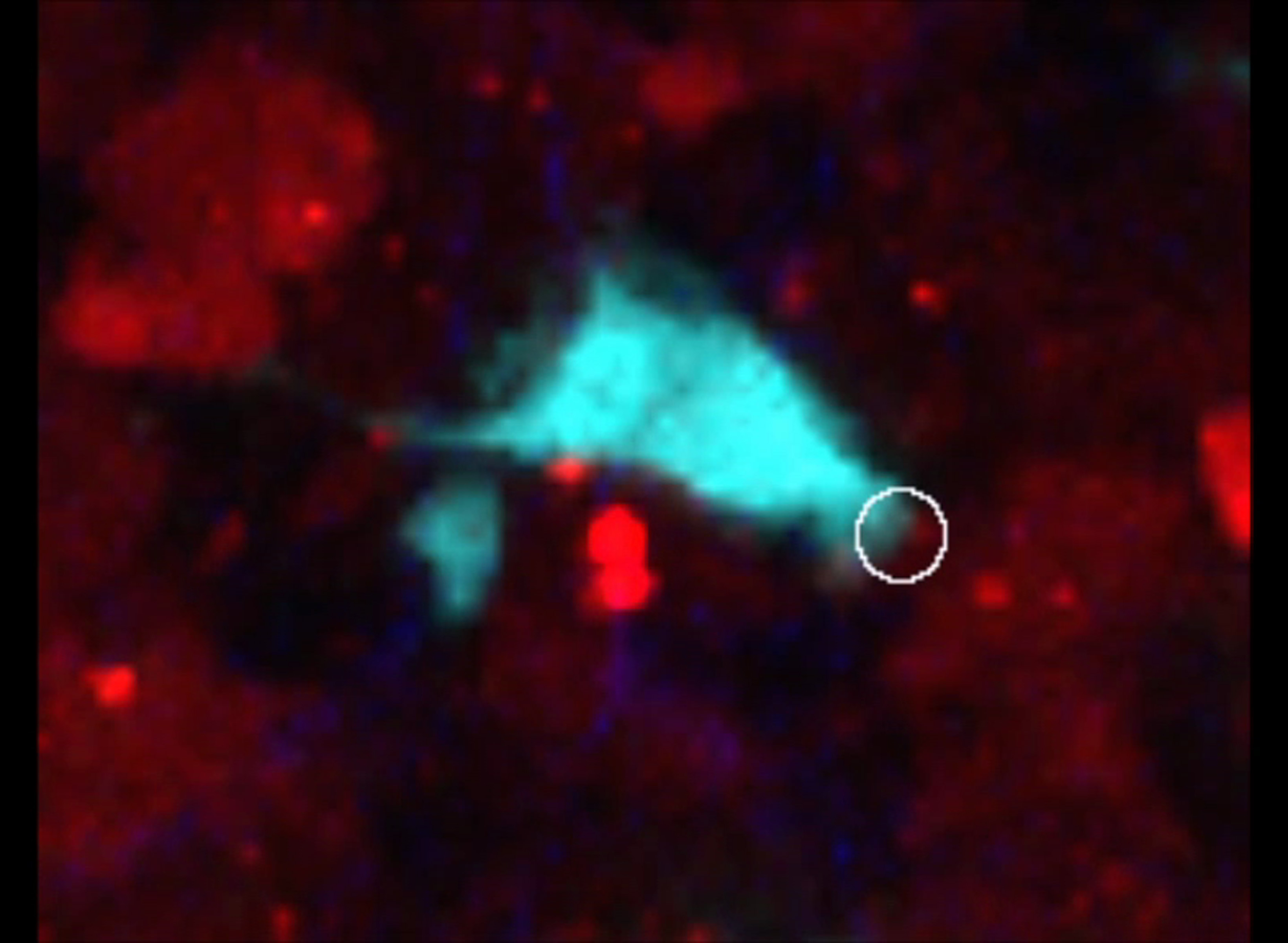

Supplement: Movie S2. Intravital Imaging Movie Example 2 Showing the Release of an Extracellular Vesicle, Related to Figure 1 — Shown is an intravital movie that was acquired of a MDA-MB-231 tumor consisting of cells expressing different fluorescent proteins. All frames in the movie represent maximum projections of 5 z planes with a total z volume of 25 μm. The cell that releases an extracellular vesicle is indicated in cyan, and the released EV is marked with a circle. [file mmc5.jpg]

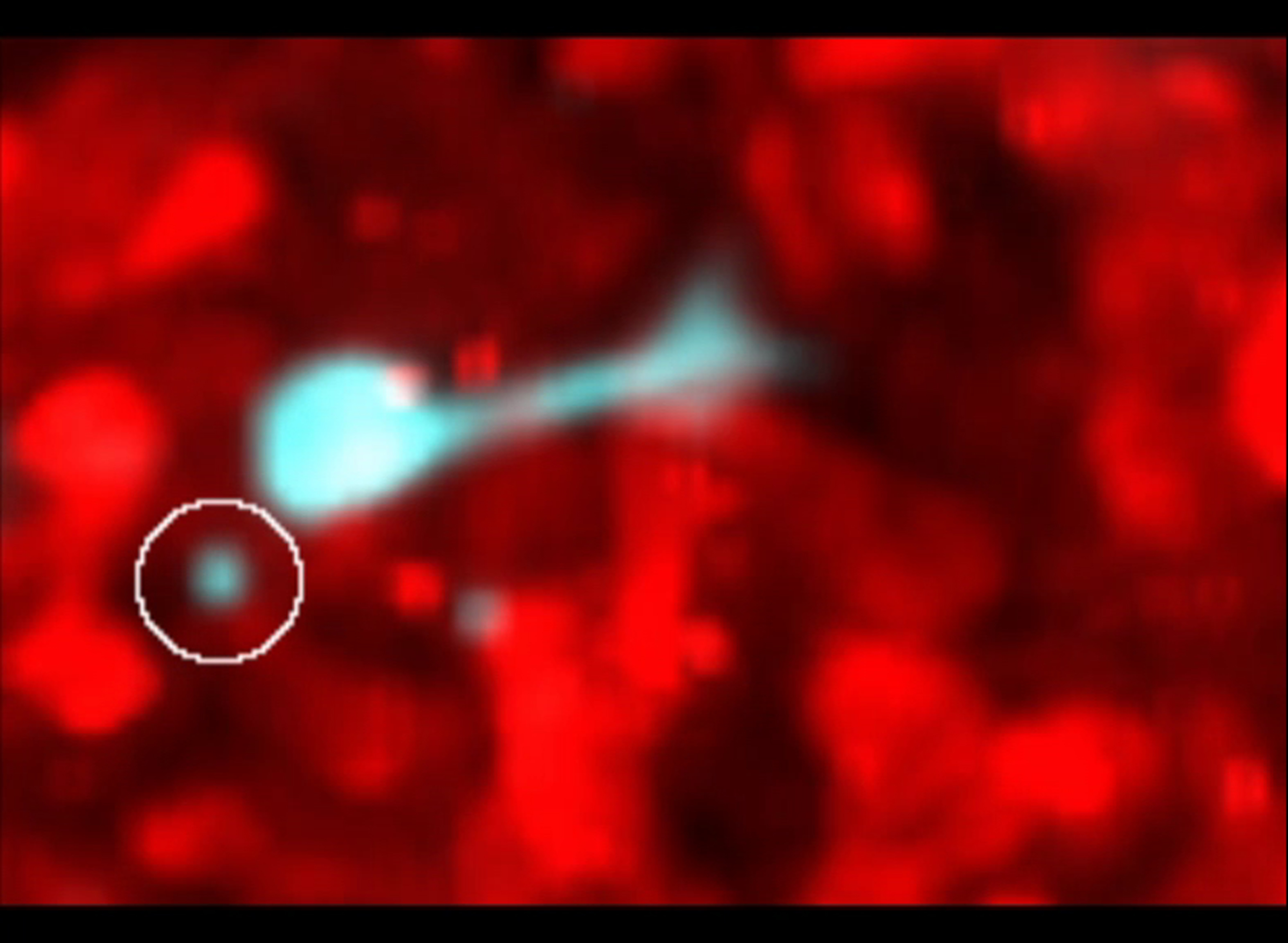

Supplement: Movie S3. Intravital Imaging Movie Example 3 Showing the Release of an Extracellular Vesicle, Related to Figure 1 — Shown is an intravital movie that was acquired of a MDA-MB-231 tumor consisting of cells expressing different fluorescent proteins. All frames in the movie represent maximum projections of 5 z planes with a total z volume of 25 μm. The cell that releases an extracellular vesicle is indicated in cyan, and the released EV is marked with a circle. [file mmc6.jpg]

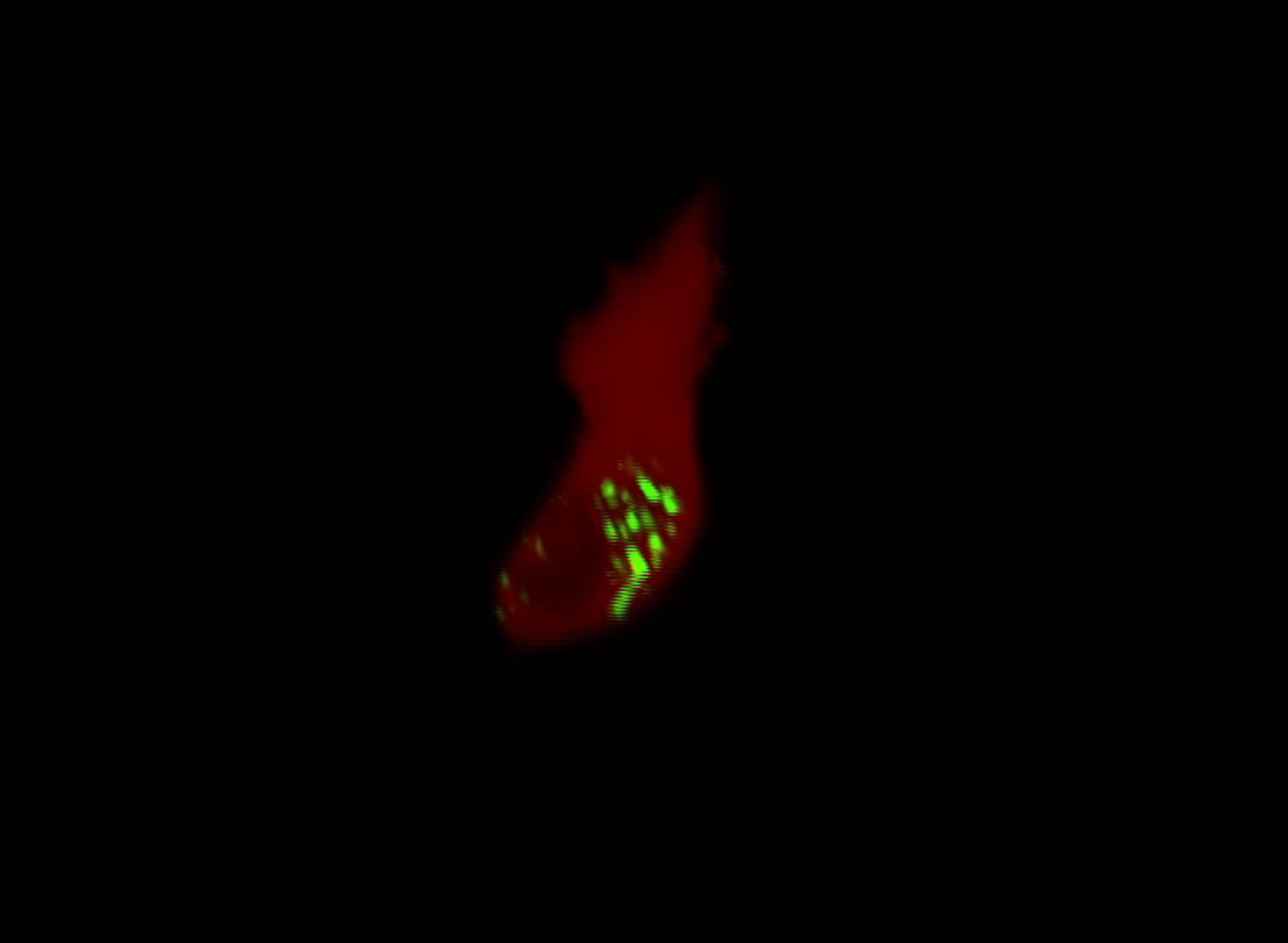

Supplement: Movie S4. Three-Dimensional Movie of a Tumor Cell that Has Taken Up Extracellular Vesicles, Related to Figure 2 — A three-dimensional movie of a cell (red) that has taken up PKH67-labeled MDA-MB-231-isolated extracellular vesicles (green). [file mmc7.jpg]

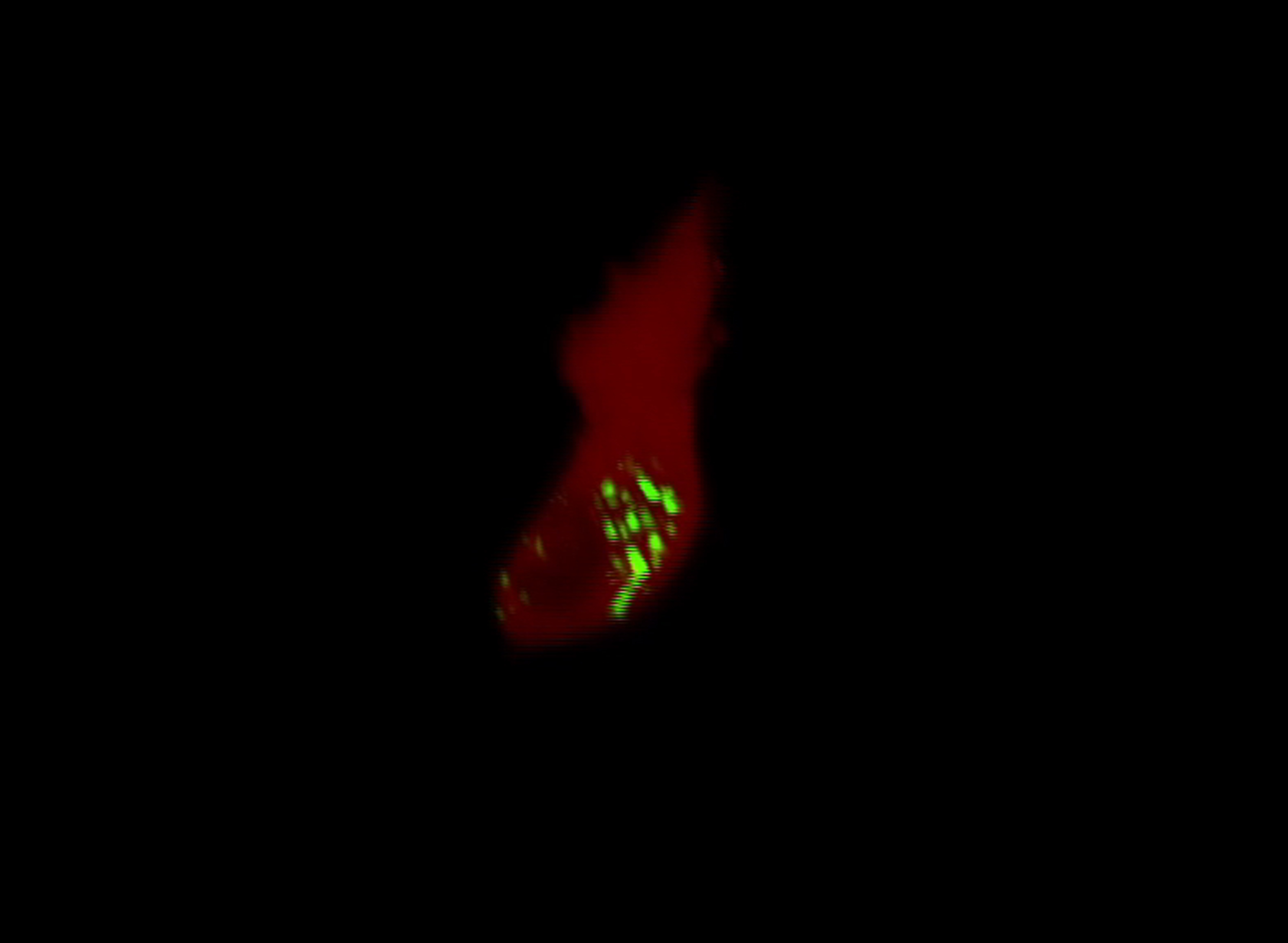

Supplement: Movie S5. Three-Dimensional Reconstruction of an Intravitally Imaged MDA-MB-231 Tumor Reporting EV Transfer, Related to Figure 4 — Three-dimensional movie of an intravital z stack (620 μm × 620 μm × 250 μm) of a tumor consisting of Cre+ CFP+ MDA-MB-231 cells and reporter+ MDA-MB-231 cells. Scale bar represents 50 μm. [file mmc8.jpg]
