# Supplementary material for: In Vivo Imaging Reveals Extracellular Vesicle-Mediated Phenocopying of Metastatic Behavior
Source: Cell. 2015 May 21;161(5):1046–57. doi: 10.1016/j.cell.2015.04.042 (PMC4448148; doi:10.1016/j.cell.2015.04.042)
Supplement: Document S2. Article plus Supplemental Information [file mmc9.pdf]

Cell

Article

# In Vivo Imaging Reveals Extracellular Vesicle-Mediated Phenocopying of Metastatic Behavior

## Graphical Abstract

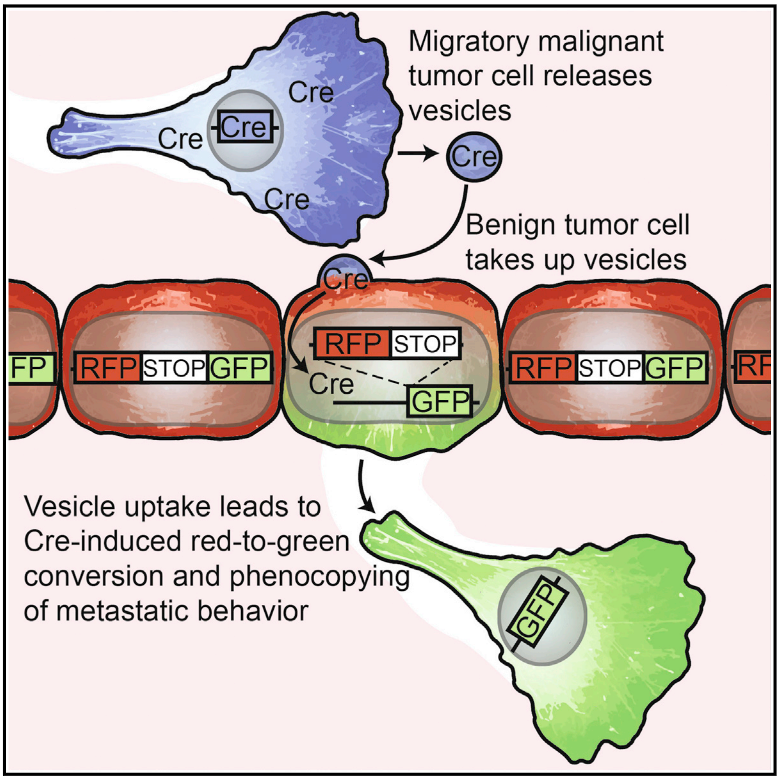


## Authors

Anoek Zomer, Carrie Maynard, ..., Dirk Michiel Pegtel, Jacco van Rheenen

## Correspondence

j.vanrheenen@hubrecht.eu

## In Brief

Intravital imaging experiments reveal the extracellular vesicle-mediated exchange of molecules important for metastasis between tumor cells in living mice. This exchange results in metastatic properties being conferred to the receiving cell.

## Highlights

- Visualization of extracellular vesicle exchange between tumor cells in living mice
- Direct in vivo evidence of local and systemic mRNA exchange between tumor cells
- The exchange of biomolecules is mediated through extracellular vesicles
- Metastatic behavior can be phenocopied through extracellular vesicle exchange

## Accession Numbers

GSE66488

Zomer et al., 2015, Cell *161*, 1046–1057
May 21, 2015 ©2015 The Authors
http://dx.doi.org/10.1016/j.cell.2015.04.042

Cell

Article

# In Vivo Imaging Reveals Extracellular Vesicle-Mediated Phenocopying of Metastatic Behavior

Anoek Zomer,[1] Carrie Maynard,[1] Frederik Johannes Verweij,[2] Alwin Kamermans,[1] Ronny Schäfer,[1] Evelyne Beerling,[1] Raymond Michel Schiffelers,[3] Elzo de Wit,[1] Jordi Berenguer,[4] Saskia Inge Johanna Ellenbroek,[1] Thomas Wurdinger,[4,5] Dirk Michiel Pegtel,[2] and Jacco van Rheenen[1,*]

[1]Cancer Genomics Netherlands, Hubrecht Institute-KNAW & University Medical Center Utrecht, Uppsalalaan 8, 3584 CT Utrecht, the Netherlands

[2]Department of Pathology, VU University Medical Center, Cancer Center Amsterdam, De Boelelaan 1117, 1081 HV Amsterdam, the Netherlands

[3]Laboratory Clinical Chemistry & Haematology, University Medical Center Utrecht, Heidelberglaan 100, 3584 CX Utrecht, the Netherlands

[4]Department of Neurosurgery, VU University Medical Center, Cancer Center Amsterdam, De Boelelaan 1117, 1081 HV Amsterdam, the Netherlands

[5]Department of Neurology, Massachusetts General Hospital and Harvard Medical School, 149 13th Street, Charlestown, MA 02129, USA

*Correspondence: j.vanrheenen@hubrecht.eu

http://dx.doi.org/10.1016/j.cell.2015.04.042

This is an open access article under the CC BY license (http://creativecommons.org/licenses/by/4.0/).

## SUMMARY

Most cancer cells release heterogeneous populations of extracellular vesicles (EVs) containing proteins, lipids, and nucleic acids. In vitro experiments showed that EV uptake can lead to transfer of functional mRNA and altered cellular behavior. However, similar in vivo experiments remain challenging because cells that take up EVs cannot be discriminated from non-EV-receiving cells. Here, we used the Cre-LoxP system to directly identify tumor cells that take up EVs in vivo. We show that EVs released by malignant tumor cells are taken up by less malignant tumor cells located within the same and within distant tumors and that these EVs carry mRNAs involved in migration and metastasis. By intravital imaging, we show that the less malignant tumor cells that take up EVs display enhanced migratory behavior and metastatic capacity. We postulate that tumor cells locally and systemically share molecules carried by EVs in vivo and that this affects cellular behavior.

## INTRODUCTION

The heterogeneous nature of tumors dramatically complicates the successful treatment of cancer (Meacham and Morrison, 2013). Tumors display intratumoral heterogeneity as a consequence of genetic differences between individual cells. Together with the diverging microenvironment surrounding tumor cells, this leads to intercellular differences in epigenetic profiles and, subsequently, to differential cellular behavior (Bissell and Hines, 2011; Postovit et al., 2006). The tumor microenvironment consists of cells, soluble factors such as growth factors, and non-soluble factors, including the extracellular matrix (Joyce and Pollard, 2009). A growing number of studies suggest that extracellular vesicles (EVs) may also be an important microenvironmental factor (Raposo and Stoorvogel, 2013) that could potentially affect tumor heterogeneity. Many different cell types have been shown to transfer biomolecules, including proteins, lipids, and nucleic acids, through the release and uptake of EVs (Raposo and Stoorvogel, 2013). In vitro assays showed that EV cargo, such as mRNA, is functional in cells that take up EVs (Valadi et al., 2007), leading to behavioral changes of recipient cells (Ratajczak et al., 2006; Salomon et al., 2013). Tumor cells also release a wide variety of EVs (Bobrie and Théry, 2013; Théry et al., 2009), the cargo of which can potentially be used as biomarkers (Balaj et al., 2011; D'Souza-Schorey and Clancy, 2012; Skog et al., 2008). The cargo of EVs isolated from tumor cell culture media can be bioactive because injection of concentrated tumor-cell-line-derived EVs educates tumor-supporting cells, such as bone-marrow-derived cells, that subsequently prepare sentinel lymph nodes and lungs for metastasis (Hood et al., 2011; Peinado et al., 2012). Vice versa, EVs from stromal cells—for example, fibroblasts and activated T cells—have been shown to alter the protrusive and migratory behavior of tumor cells (Cai et al., 2012; Luga et al., 2012).

EV exchange between tumor cells in heterogeneous tumors could potentially dramatically accelerate tumor progression if highly metastatic cells spread their malignant behavior to less malignant cells. However, acquiring direct evidence for the exchange of active biomolecules between tumor cells in vivo so far remained challenging due to many technical limitations (Raposo and Stoorvogel, 2013; Théry, 2011). First, in vivo tumor cells are exposed to EVs released by various cell types, making it impossible to specifically measure the effect of tumor-derived EVs on other tumor cells. To address this, concentrated EV preparations isolated from cancer cell culture supernatants have been injected into animal models. However, this does not reflect the location, concentration, and continuous nature of EV release

by tumor cells in their in vivo setting. Second, techniques have been lacking to directly track and study cells that take up in-vivo-released tumor EVs and to compare their behavior to counterparts that did not take up tumor-released EVs. Therefore, it is unknown whether, in heterogeneous tumors in living mice, cells with high metastatic potential can transfer biomaterial to less metastatic cells, thereby influencing tumor progression.

Here, we combine high-resolution intravital imaging with a Cre recombinase-based method to study EV exchange between tumor cells. To show the spread of metastatic behavior through EVs in living mice, we directly visualized the release of EVs by highly metastatic human MDA-MB-231 mammary tumor cells. Moreover, in living mice, we identified and studied the behavior of less malignant human T47D mammary tumor cells that take up these in-vivo-released EVs by more malignant MDA-MB-231 cells. Our study illustrates that tumor heterogeneity contains an additional layer of complexity with tumor cells sharing biomolecules through local and systemic transfer of EVs, which profoundly affects cell behavior.

## RESULTS AND DISCUSSION

### Tumor Cells Release a Heterogeneous Population of EVs In Vivo

To examine the in vivo release of EVs by highly metastatic tumor cells, we orthotopically transplanted MDA-MB-231 cells in the mammary glands of mice, leading to the formation of highly metastatic mammary tumors. Next, we examined sections of these tumors using confocal microscopy. As expected, we observed EVs with a wide variety of sizes (Figure 1A). Next, to further analyze the in vivo release of these EVs, we intravitally imaged similar mammary tumors consisting of differently labeled MDA-MB-231 cells (Figure 1B). We observed the release of EVs with a diameter of 2.5 $\mu$m and smaller (Figure 1B and Movie S1, S2, and S3). Because the resolution of a multiphoton microscope is one order of magnitude smaller than the size of EVs such as exosomes ($\sim$500 versus 50 nm, respectively), the signal of small EVs will be blurred to an area according to the microscope's point spread function (>1 $\mu$m diameter). To characterize the heterogeneous mixture of EVs, including plasma membrane-shed vesicles and exosomes, we isolated EVs from MDA-MB-231 tumors as confirmed by electron microscopy (Figure 1C) and determined the size distribution by nanoparticle tracking analysis (NanoSight) (Figure 1D). Next, to identify potentially functional EV cargo, we took the cells and EVs from tumors and isolated their mRNA. We identified the differential mRNA profile using gene expression arrays (Figure 1E) and found a significant enrichment of mRNA molecules from >200 genes in EVs compared to the cells (red dots Figure 1E and Table S1). Gene ontology analysis showed that many of these enriched mRNA molecules are involved in migration and metastasis (indicated in red in Figure 1F and Table S2), both of which promote tumor progression. Which mRNAs carried by EVs are critical for induction of behavioral changes of recipient cells depends on the expression profile of the recipient cell. The mRNAs enriched in EVs released by MDA-MB-231 cells may change the behavior of MDA-MB-231 cells that take up these EVs due to the abundance of mRNAs involved in metastasis. However, mRNAs from MDA-MB-231 cells that are not vesicular enriched but abundantly present may induce behavioral changes when the mRNA level of those particular genes is low in other recipient cell types. This also holds true for other potentially functional biomolecules loaded in EVs such as DNA, (signaling) proteins, lipids, and microRNAs (Raposo and Stoorvogel, 2013). Therefore, it is important to realize that our data do not point to individual biomolecules that induce phenotypic changes in recipient cells but illustrate that, within the tumor microenvironment, a heterogeneous population of EVs is present containing biomolecules, among which there are mRNAs that are involved in migration and metastasis.

### The Cre/LoxP System Can Be Used to Study EV Uptake

To study EV exchange in vivo, we utilized the Cre-LoxP system to induce a color switch specifically in reporter-expressing cells (reporter$^{+}$) that take up EVs released from cells expressing the Cre recombinase (Cre) (Cre$^{+}$ cells) (Figure 2A). This Cre-LoxP system uses the reverse approach of rendering donor cells vesiculation deficient; recipient cells that do not take up EVs are marked with DsRed (i.e., unrecombined reporter) and serve as an internal control for eGFP-expressing recipient cells that have taken up EVs. Importantly, the detection is restricted to uptake of EVs released by Cre$^{+}$ cells. This enables the analysis of biological effects induced by EVs released from an a priori defined tumor cell population in vivo.

To study EV exchange using the Cre-LoxP system, we generated a MDA-MB-231 cell line expressing CFP and Cre. We isolated EVs released from this Cre$^{+}$ cell line, as confirmed by electron microscopy (Figure 2B), and determined their size distribution (Figure 2C). Importantly, tumor cells were able to take up purified and fluorescently labeled EVs in vitro (Figure 2D and Movie S4). Next, by western blot analysis, we confirmed that the EVs contained the typical EV markers CD63 and Hsp70. Although Cre protein was undetectable (Figure 2E) in EVs, Cre mRNA was readily detected by RT-PCR (Figure 2F). Thus, Cre mRNA molecules are incorporated into EVs that can be taken up by tumor cells.

In order to determine whether Cre from CFP$^{+}$ Cre$^{+}$ cells can be functionally transferred to reporter$^{+}$ cells, we co-cultured MDA-MB-231 Cre$^{+}$ and reporter$^{+}$ cells for 1 week. In co-cultures of Cre$^{+}$ cells and reporter$^{+}$ cells, converted eGFP$^{+}$ reporter$^{+}$ cells appeared (Figure 3A), and the percentage of cells that switched color increased with the ratio of Cre$^{+}$ cells over reporter$^{+}$ cells (Figure 3B). Importantly, nearly all eGFP$^{+}$ cells were negative for CFP, excluding the possibility that cell fusion between Cre$^{+}$ and reporter$^{+}$ cells, rather than EV transfer, causes the appearance of eGFP$^{+}$ cells (Figure 3C). To further investigate cell-cell contact-independent biomolecule transfer as a mechanism of functional Cre transfer, we used transwell co-cultures that preclude passage of Cre$^{+}$ cells but allow passage of Cre$^{+}$ EVs. Indeed, seeding of Cre$^{+}$ cells in the top well led to the appearance of eGFP$^{+}$ reporter$^{+}$ cells in the lower well (Figure 3D, bottom). To study whether malignant MDA-MB-231 cells can also transfer Cre to other, less malignant, tumor cells, we generated T47D and MCF-7 reporter$^{+}$ cells. Interestingly, Cre was also transferred from malignant to these less malignant cells in a cell-cell contact-independent manner (Figure 3D, middle and

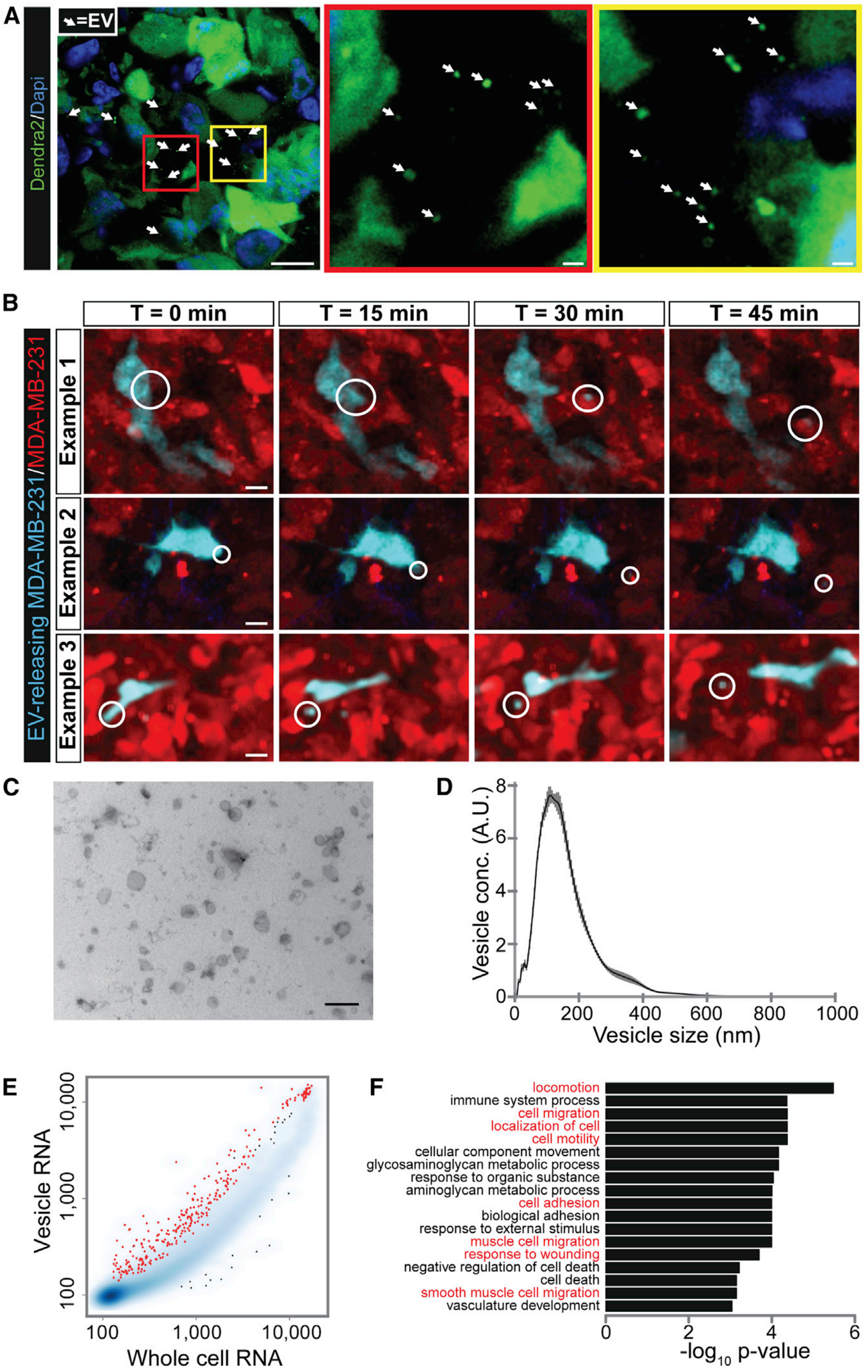


**Figure 1. In Vivo Visualization of EV Release and Characterization of the mRNA EV Cargo**

(A) Images of MDA-MB-231 tumor sections. Boxed areas are shown on the right. Arrows point to different EVs. Images were gamma adjusted to also show dim and small EVs. Scale bars represent 10 μm (left image) and 1 μm (middle and right image).

(B) Stills from intravital imaging movies (Movies S1, S2, and S3) of MDA-MB-231 tumors where CFP-marked MDA-MB-231 cells surrounded by DsRed-marked cells release EVs (marked by circles). Images are maximum projections of 5 z planes with a total z volume of 25 μm. Scale bars represent 10 μm.

(C) Electron microscopy image of EVs isolated from MDA-MB-231 tumors. Scale bar represents 500 nm.

(D) NanoSight particle analysis displaying the size distribution of EVs isolated from MDA-MB-231 tumors. Black line represents mean of five experiments, SD in gray.

(E) mRNA profile of EVs and cells isolated from MDA-MB-231 tumors. Smoothed color density representation shows RNA levels of whole cells versus RNA derived from EVs. Red dots represent genes that are significantly and more than 2-fold enriched in EVs; significance is based on replicate experiments (n = 6).

(F) These genes in (D) were used as input for WebGestalt analysis (Wang et al., 2013). Bar plot representation of p values from a gene ontology enrichment analysis. Biological processes involved in cell movement are shown in red.

See also Movies S1, S2, and S3 and Tables S1 and S2.

right) and concentration-dependent manner (Figure 3E). These data suggest that, in vitro, tumor cells with a high metastatic potential can functionally transfer biomolecules (e.g., Cre) to less malignant cells that could potentially affect their physiology.

## Tumor Cells Take Up Tumor-Cell-Derived Cre$^+$ EVs In Vivo

Next, we used the Cre-LoxP system to investigate the in vivo existence of cell-to-cell transfer of mRNA and other biomolecules in different mammary and melanoma tumor models (Figures 4 and S1). We confirmed that EVs isolated from Cre$^+$ tumors contained Cre mRNA (Figure 4A). Next, we pretreated these EVs with RNase and proteinase K (to degrade free-floating Cre RNA and Cre protein) and showed that, upon intratumoral injection, reporter$^+$ cells take up these EVs and report Cre activity (Figure 4B). Thus, cells only report vesicular Cre, which is further supported by our observation that cells do not report the uptake of free-floating Cre upon injection of recombinant Cre protein (Figure 4C) or whole-cell lysate of a Cre$^+$ tumor (Figure 4D). Interestingly, similar to our in vitro observations, in all mammary and melanoma tumor models that we studied, eGFP$^+$ reporter$^+$ cells (negative for CFP [Figure S2]) appeared in tumors consisting of Cre$^+$ cells and reporter$^+$ cells (Figure 4E and see Movie S5 for a 3D reconstruction of a z stack of intravital images). Combined, these data suggest that in vivo tumor cells can release and take up EVs and that the functional mRNA transfer via EVs can be reported by the Cre-LoxP system.

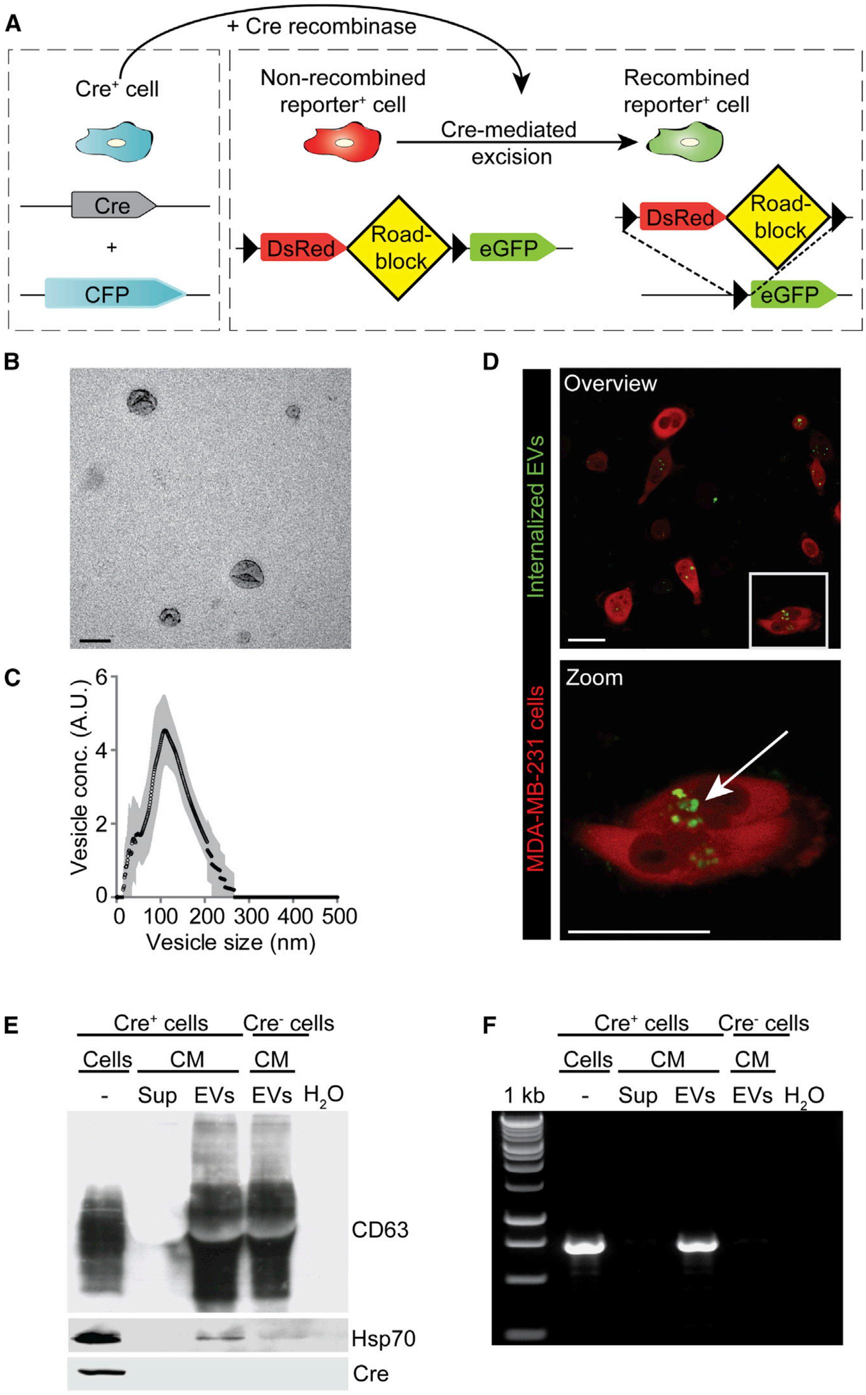


**Figure 2. The Cre-LoxP System to Measure EV Uptake**

(A) Cartoon showing the Cre-LoxP system used to report the transfer of Cre recombinase (Cre) activity. A red-to-green color switch is induced in reporter+ cells (right) upon the transfer of Cre activity derived from CFP+ Cre+ cells (left).

(B) Electron microscopy analysis of EVs isolated from MDA-MB-231 cell cultures. Scale bar represents 150 nm.

(C) NanoSight particle analysis displaying the size distribution of EVs isolated from MDA-MB-231 cell cultures. Black line represents mean of five experiments, SD in gray.

(D) Confocal images of reporter+ cells that have taken up green-labeled MDA-MB-231-isolated EVs 3 hr after addition of these EVs to the culture. The boxed area is shown on below the image. Arrow points to EVs that are taken up. Scale bars represent 50 μm.

(E and F) Characterization of EVs isolated from MDA-MB-231 conditioned media (CM) using high-speed centrifugation. The supernatant (sup) obtained during this centrifugation procedure was used as negative control in (E) western blot analysis of CD63, Hsp70, and Cre protein levels and (F) RT-PCR for Cre mRNA of samples as indicated.

See also Movie S4.

## EV Exchange between Tumor Cells and Non-Tumor Cell Types

Our Cre-Lox reporter enables us to study the uptake of EVs released by defined populations of cells. Therefore, in our in vivo syngeneic melanoma tumor model, we tested whether different types of non-tumor cells take up tumor-released EVs and whether tumor cells can take up EVs released from non-tumor cells. We generated Cre-expressing B16 melanoma cells and injected these cells into mice ubiquitously expressing the Cre-LoxP reporter tdTomato (tdTomato B6 mice) (Figure 5A). Within 2 weeks, these mice developed palpable tumors, and we examined various tissues for the presence of different types of non-tumor cells that report the uptake of Cre (i.e., tdTomato expression). Interestingly, we observed non-tumor cells expressing tdTomato in all examined tissues (tumors, lymph nodes, lungs, and spleens) (Figure 5B). To investigate the nature of these cells, we analyzed the expression of different markers, including CD45, which is a general immune cell marker. Interestingly, we observed CD45− and CD45+ cells, suggesting that both non-immune cells and immune cells have taken up tumor-released EVs (Figure 5C). By immunohistochemistry, we identified various cell types, including Gr1+ cells and F4/80+ cells, suggesting the transfer of tumor-released EVs to neutrophils and macrophages (Figure 5D). From this, we conclude that not only tumor cells but also multiple non-tumor cell types are able to take up tumor cell-released EVs.

To test whether tumor cells can take up EVs released from non-tumor cells, we generated B16 melanoma cells that express the Cre-LoxP reporter as shown in Figure 2A. Next, we injected these reporter cells into B6 mice in which Cre is ubiquitously

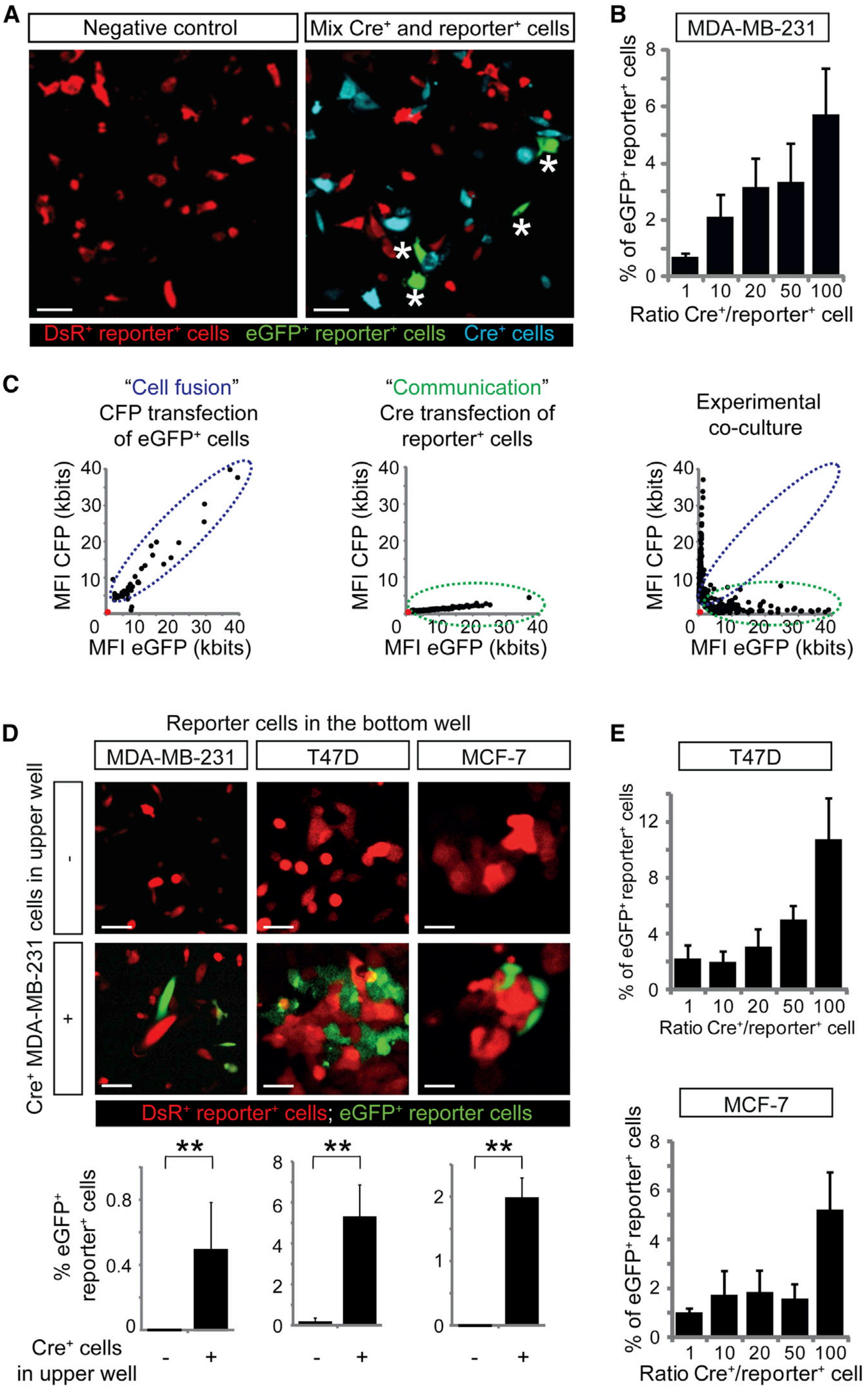


**Figure 3. In Vitro Visualization of Transfer of Cre Activity**

(A) Images of MDA-MB-231 cultures consisting of reporter+ cells (red) only or a mixture of Cre+ (cyan) and reporter+ cells. Note the appearance of eGFP+ reporter+ cells in the presence of Cre+ cells, indicated by asterisks. Scale bars represent 50 μm.

(B) Quantification of the percentage of eGFP+ MDA-MB-231 cells in several conditions with varying ratios of Cre+ and reporter+ cells (n = 5 independent experiments). Data are represented as mean ± SEM.

(C) For 50 cells, the mean fluorescence intensity (MFI) of eGFP (reporting Cre activity) is plotted against the MFI of CFP (Cre+ cells). Intensities of non-fluorescent cells are zero (red dot). In the left plot, cell fusion was experimentally simulated by transfecting eGFP+ cells with CFP DNA. In the middle plot, communication was experimentally simulated by transfecting reporter+ cells with Cre DNA. The right plot shows the results of the experimental co-culture. The blue dotted line outlines cells that show cell fusion, and the green dotted line shows eGFP expression derived from cell-cell communication other than fusion.

(D) Images of MDA-MB-231 reporter+ cells (left), T47D reporter+ cells (middle), or MCF-7 reporter+ cells (right) at the bottom well of a transwell system in the absence (upper images) or presence (lower images) of MDA-MB-231 Cre+ cells in the upper well (n = 3 independent experiments). Bar graphs below show quantifications. Data are represented as mean ± SEM. Scale bars represent 50 μm.

(E) Quantification of T47D cells (upper graph) and MCF-7 cells (lower graph) that express eGFP in several conditions with varying ratios of Cre+ and reporter+ cells (n = 5 independent experiments). Data are represented as mean ± SEM.

expressed by the ACTB promoter (Figure 5E). Within 2 weeks, these mice developed palpable tumors that we examined for the presence of tumor cells reporting the uptake of EVs released by Cre-expressing non-tumor cells. Only occasionally (2 ± 0.7 cells per $mm^3$; n = 3 mice), we observed tumor cells that report the uptake of EVs released from non-tumor cells (Figure 5F). This observation is in line with the general assumption that non-tumor cells release relatively less EVs compared to tumor cells (Balaj et al., 2011; Raposo and Stoorvogel, 2013). From this, we conclude that, although B16 melanoma cells have the ability to take up EVs from healthy cells, this transfer does not seem to happen frequently.

## The Local and Systemic Transfer of MDA-MB-231-Derived EVs to Less Malignant T47D Cells

Because tumor cells can take up EVs released by other tumor cells, we questioned whether less malignant tumor cells can take up EVs derived from malignant tumor cells by analyzing tumors of mice injected with a mixture of malignant MDA-MB-231 Cre+ cells and less malignant T47D reporter+ cells (Figure 6A, left). While the T47D cells outcompeted the MDA-MB-231 cells to some extent, the percentage of MDA-MB-231 cells in MDA-MB-231/T47D tumors was sufficient to observe local transfer of Cre activity between these cell types (Figure 6B, left). Next, we tested the possibility of systemic transfer of Cre activity by injecting MDA-MB-231 Cre+ cells

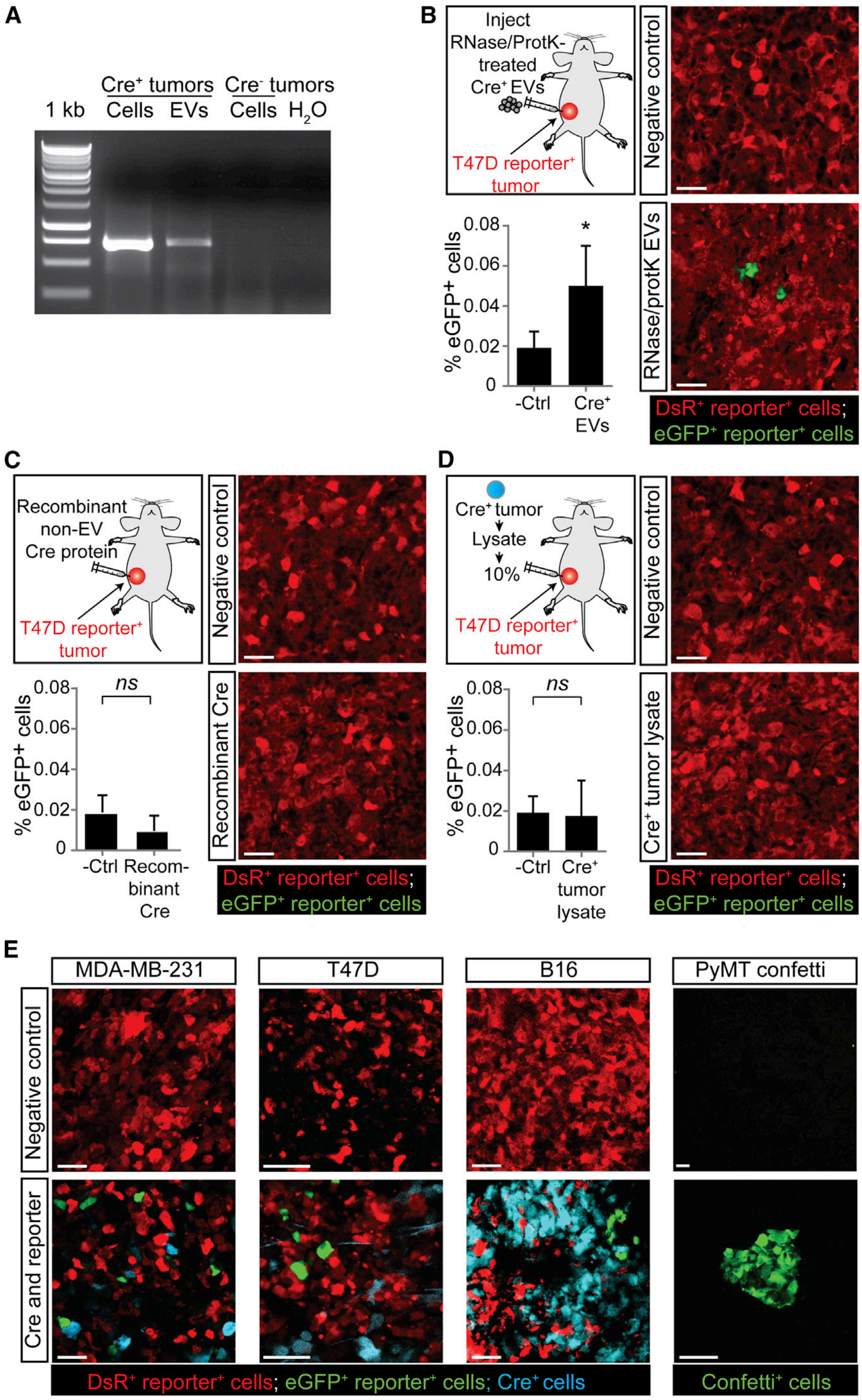


**Figure 4. The Transfer of EVs between Tumor Cells in Living Mice**

(A) Cells and EVs were isolated from $Cre^+$ tumors and $Cre^-$ tumors. Shown is the RT-PCR of indicated samples.

(B) EVs isolated from $Cre^+$ MDA-MB-231 cells and pretreated with RNase and proteinase K (protK) were intratumorally injected in T47D $reporter^+$ tumors. Shown are representative images of these tumors and quantification of $eGFP^+$ cells (n = 3 mice). Data are represented as mean ± SEM. Scale bars represent 50 μm.

(C) Recombinant Cre protein (110 ng) was intratumorally injected in T47D $reporter^+$ tumors. Shown are representative images of these tumors and quantification of $eGFP^+$ cells (n = 4 mice). Data are represented as mean ± SEM. Scale bars represent 50 μm.

(D) Whole-cell lysate of 10% of a $Cre^+$ MDA-MB-231 tumor was intratumorally injected in T47D $reporter^+$ tumors. Shown are representative images of these tumors and quantification of $eGFP^+$ cells (n = 4 mice). Data are represented as mean ± SEM. Scale bars represent 50 μm.

(E) Confocal images of tumors containing only $reporter^+$ cells in control animals (negative control) and in tumors that contain $Cre^+$ cells and $reporter^+$ cells. MDA-MB-231 mammary tumor (260 ± 67 $eGFP^+$ tumor cells per intravital 620 × 620 × 100 μm z stack, n = 3 mice [for 3D reconstruction see Movie S5]), T47D mammary tumor (513 ± 66 $eGFP^+$ tumor cells per 16-μm-thick tumor section, n = 3 mice), PyMT mammary tumor (30 ± 13 $confetti^+$ tumor cells per 20 -μm-thick tumor section, n = 3 mice), and B16 melanoma (43 ± 10 $eGFP^+$ tumor cells per 100-μm-thick tumor section, n = 3 mice). For the PyMT model, the Cre-confetti-reporter system was used (see Figures S1B and S1C; all colors are shown in green). Scale bars represent 50 μm.

See also Movie S5 and Figures S1 and S2.

and T47D $reporter^+$ cells in contralateral mammary glands (Figure 6A, right). We observed that T47D cells reported Cre activity derived from MDA-MB-231 $Cre^+$ cells even when injected in contralateral tumors (Figure 6B, right). As expected, systemic transfer of Cre activity was less efficient than local transfer (Figure 6C). However, the observed efficiency in local transfer presumably represents an underestimation of the actual transfer because Cre-reporting efficiency depends on the ratio of $Cre^+$ over $reporter^+$ cells (Figures 3B and 3E), which was 20 to 100 times smaller in the local communication experiment compared to the distant communication experiment. In addition, the observed efficiency in systemic transfer may be slightly over-represented by a few Cre cells that have migrated toward this distant area (reseeding) and thereby potentially transfer EVs locally (23 ± 10 cells per $mm^3$, n = 3 mice). However, the number of reseeded $Cre^+$ cells is negligible compared to the total number of EV-producing $Cre^+$ cells present at the contralateral side. To confirm the distant EV transfer in a less aggressive tumor model in which reseeding events are less likely, we analyzed contralateral MCF-7 $Cre^+$ and T47D $reporter^+$ tumors (Figure S3). In this model, we did not observe any $Cre^+$ MCF-7 cells in the contralateral T47D $reporter^+$ tumors (n = 4 mice), whereas T47D $reporter^+$ cells report the uptake of EVs

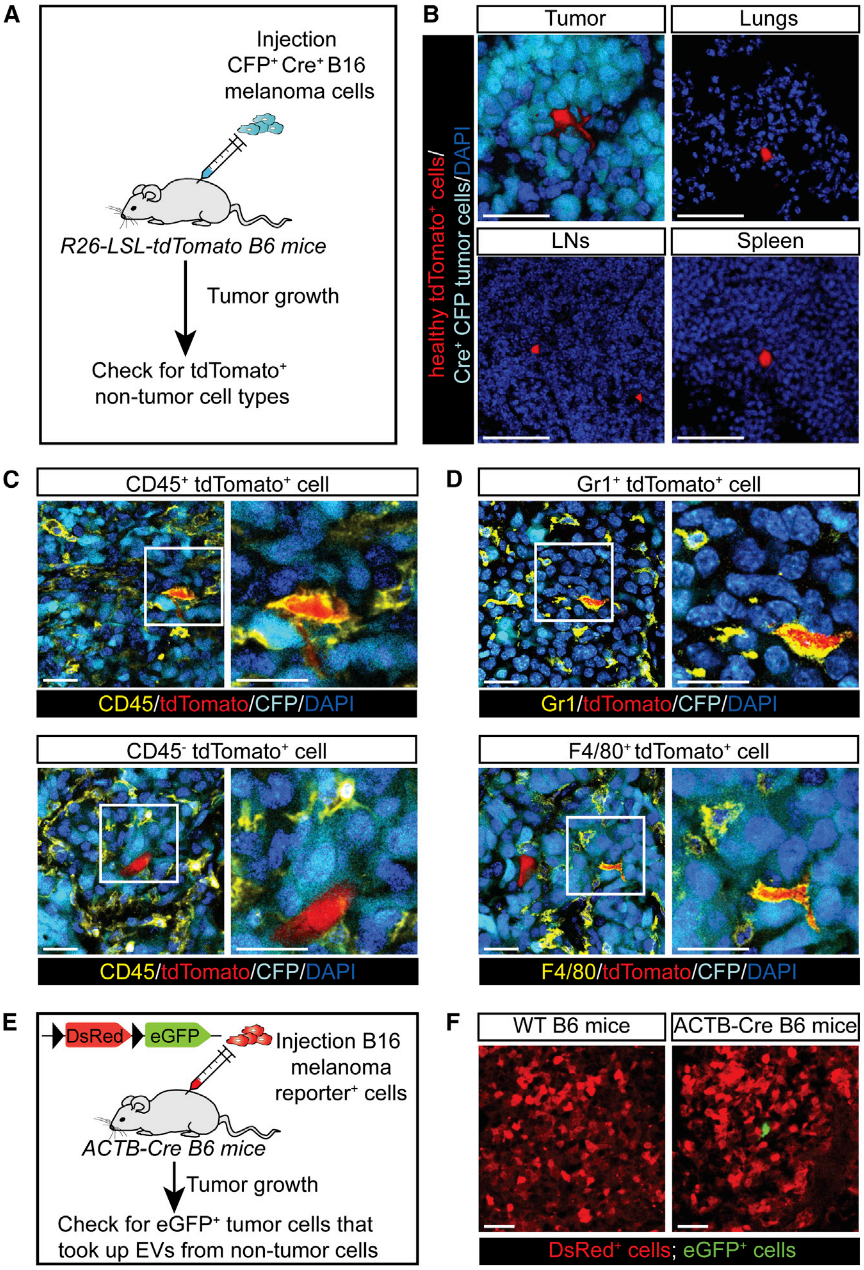


**Figure 5. Transfer of EVs between Tumor and Healthy Cells in Living Mice**

(A) B16 melanoma CFP$^+$ Cre$^+$ cells were injected subcutaneously in Cre reporter B6 mice ubiquitously expressing a floxed-STOP-floxed-tdTomato (R26-LSL-tdTomato mice).

(B) Representative confocal images (n = 3 mice) showing tdTomato$^+$ cells in indicated tissues (tumors 374 ± 76 cells/mm$^3$, lungs 69 ± 17 cells/mm$^3$, lymph nodes (LNs) 168 ± 69 cells/mm$^3$, spleens 104 ± 22 cells/mm$^3$). CFP is indicated in cyan, tdTomato is indicated in red, and DAPI is indicated in blue. Scale bars represent 50 μm. All numbers represent mean ± SEM.

(C) Confocal images of CD45$^+$ (top images) and CD45$^-$ tdTomato$^+$ (bottom images) cells found in the mice described in (A). Boxed areas are shown on the right. CD45 staining is indicated in yellow, tdTomato is indicated in red, CFP is indicated in cyan, and DAPI is indicated in blue. Scale bars represent 25 μm.

(D) Confocal images show Gr1$^+$ cells (top images) and F4/80$^+$ tdTomato$^+$ (bottom images) cells found in the mice described in (A). Boxed areas are shown on the right. Gr1(top) and F4/80 (bottom) stainings are indicated in yellow, tdTomato is indicated in red, CFP is indicated in cyan, and DAPI is indicated in blue. Scale bars represent 25 μm.

(E) B16 melanoma reporter$^+$ cells were injected subcutaneously in B6 ACTB-Cre mice that express Cre ubiquitously.

(F) Tumors were isolated from ACTB-Cre mice bearing B16 melanoma reporter$^+$ tumors (n = 3 mice). Representative confocal images show eGFP$^+$ melanoma cells (2.5 ± 0.6 cells/mm$^3$). Scale bars represent 50 μm. All numbers represent mean ± SEM.

released by the MCF-7 cells located at the contralateral side (Figure S3A). Thus, both in in vitro culture models and in living mice, tumor cells release and exchange functional biomolecules (e.g., Cre) locally and systemically through EVs.

## Migration of T47D Cells Is Enhanced upon Uptake of MDA-MB-231-Derived EVs

Because we found mRNAs from many genes involved in migration and metastasis in MDA-MB-231-isolated EVs (Figure 1F) and in vitro data have shown that mRNA can be functionally transferred to other cells (Valadi et al., 2007) (as we confirmed in vivo for Cre), we investigated whether these EVs can affect the migratory behavior of T47D recipient cells in vivo. We intravitally imaged T47D cells that reported (eGFP$^+$) or did not report (DsRed$^+$) the local or distant transfer of Cre activity (Figure 6A). We tracked the migration of all individual eGFP$^+$ cells and randomly picked proximate DsRed$^+$ cells within the same imaging field (Figure 6D) and measured the average migration speed of these cells per imaging field (Figure 6E, the values of measurements of eGFP$^+$ and DsRed$^+$ cells within the same imaging field are connected with a line). In agreement with intravital observations by others (Condeelis and Segall, 2003; Joyce and Pollard, 2009; Ellenbroek and van Rheenen, 2014), migration varied among the different imaging fields and mice, presumably caused by diverse microenvironments (Figures 6E and 6F). The proximate presence of MDA-MB-231 cells favored a migration-inducing microenvironment for T47D cells because both eGFP$^+$ and DsRed$^+$ T47D reporter$^+$ cells migrated faster when MDA-MB-231 were in close proximity (local communication; Figure 6E) than when MDA-MB-231 cells were located in a distant tumor (distant communication; Figure 6F).

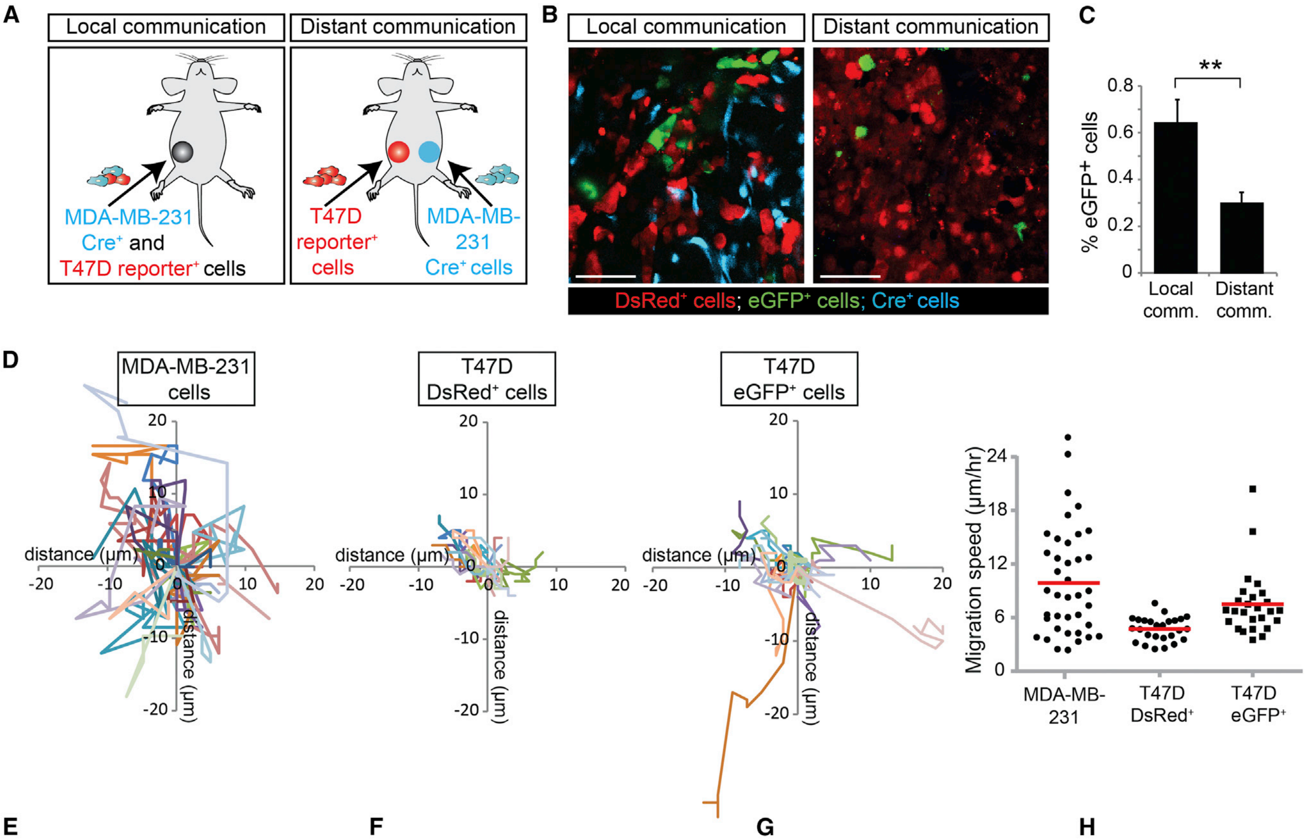


**Figure 6. Less Malignant T47D Cells that Take Up MDA-MB-231 EVs Display Enhanced Migration**

(A) MDA-MB-231 CFP$^+$ Cre$^+$ cells and T47D reporter$^+$ cells were injected as a mixture in one mammary gland (local communication) or separately in contralateral mammary glands (distant communication).

(B) Representative intravital images of the tumors in mice as indicated. Scale bars represent 50 μm.

(C) Quantification of eGFP$^+$ cells in the local and distant communication experiments (n = 26 frozen tumor sections from 3 mice). Data are represented as mean ± SEM.

(D) Mammary tumors containing MDA-MB-231 Cre$^+$ cells and T47D reporter$^+$ cells were intravitally imaged for 3 hr. Shown are migration paths of MDA-MB-231 cells and T47D DsRed$^+$ (left) and eGFP$^+$ reporter cells (right) within the same imaging field. In the far right, the migration speed of the same cells is plotted. Red lines indicate means (n > 25 cells).

(E and F) The in vivo migration speed of T47D eGFP$^+$ and DsRed$^+$ reporter$^+$ cells in tumors that report local (E) or distant (F) transfer of Cre from MDA-MB-231 Cre$^+$ cells. Shown are the average values per imaging field. Values for eGFP$^+$ and DsRed$^+$ cells in the same imaging field are connected with a line (n = 5–6 imaging fields per mouse for 3 independent mice).

(G and H) The normalized in vivo migration speed of T47D eGFP$^+$ and DsRed$^+$ reporter$^+$ cells in tumors reporting the local (G) or distant (H) transfer of Cre from MDA-MB-231 Cre$^+$ cells. Per imaging field, the values are normalized to the migration speed of neighboring DsRed$^+$ cells (n = 17–18 imaging fields in 3 mice). Data are represented as mean ± SEM.

See also Figures S3, S4, and S5.

In order to test how the transfer of biomolecules, as reported by our Cre reporter, affects migration within one space-confined microenvironment, we compared the migration of eGFP$^+$ and DsRed$^+$ cells within one imaging field. Because the eGFP$^+$ and DsRed$^+$ cells experience the same microenvironment such as cytokines within one imaging field, differential behavior between

eGFP+ and DsRed+ cells illustrates the effect of EV uptake. In all 18 distinct positions reporting local transfer (n = 3 mice) (Figure 6E) and in 15 of the 17 distinct positions reporting systemic transfer (n = 3 mice) (Figure 6F), we observed that eGFP+ cells are more migratory than neighboring DsRed+ cells. To quantify this effect within the various microenvironments, we normalized the migration distance of tumor cells to the average migration path of DsRed+ cells that did not report Cre activity in each imaging field independently (Figures 6G and 6H). Over time, we tracked the position of more than 360 cells per condition (n = 3 mice) and found that eGFP+ cells migrate 1.57 ± 0.084 (Figure 6G) and 2.37 ± 0.431 (Figure 6H) times faster than the DsRed+ cells after local and distant communication, respectively. Similar results were found in in vitro studies in which we imaged local (co-culture) and distant (transwell) communication (Figures S4A and S4B). The observed effects on migration were not due to the expression of the different fluorophores because cells that became eGFP+ upon transfection with a Cre-encoding DNA plasmid did not migrate faster than DsRed+ cells (Figure S4C). Our data suggest that tumor cell migration is dependent on non-systemic cues in the local microenvironment and that this migration can be significantly enhanced by the uptake of migration-associated mRNAs through EV transmission.

The correlation between Cre uptake and increased motility is fully consistent with the migratory-inducing mRNA cargo that we identified in MDA-MB-231-released EVs, unless motile cells are somehow more prone to take up biomolecules than less-motile cells. Several lines of evidence exclude the latter hypothesis. First, within one cell line (MDA-MB-231), the fast-migratory cells did not take up more fluorescently labeled EVs than slow-migratory cells (Figure S5A). Second, if motile cells would have a higher a priori propensity to report Cre activity, then eGFP+ cells would always migrate faster than DsRed+ cells, which was not observed when comparing the migration speed of eGFP+ and DsRed+ cells across different imaging fields (Figures 6E and 6F). Third, the percentage of cells that report Cre was not increased in imaging fields with migratory-supporting microenvironments where faster average migration was observed (Figure S5B). Fourth, the uptake of EVs produced by a less migratory cell line (MCF-7) (Figure S3B) did not lead to enhanced migration of eGFP+ cells; instead, we observed a significant decrease in migration (Figure S3C). Therefore, our data collectively show that migration of T47D cells is enhanced upon the uptake of EVs released by MDA-MB-231 cells.

## T47D Cells that Take Up MDA-MB-231 EVs Become More Metastatic

Because activating migration and invasion is one of the hallmarks of cancer (Hanahan and Weinberg, 2000, 2011) and key to metastasis (Nguyen et al., 2009), the transfer of biomolecules, including mRNAs involved in migration, may affect not only the migratory behavior but also the metastatic potential of tumor cells. To investigate this, we isolated the lungs of four mice and analyzed in total >1,000 metastases (Figure 7A). When the potential to metastasize to the lungs is enhanced by EV uptake in the population of cells that report Cre activity (eGFP+), the ratio of eGFP+ over DsRed+ cells that arrive and grow out in the lung is expected to be increased compared to the ratio of these populations of cells in the corresponding primary tumor (Figure 7A). As expected, T47D cells that take up EVs released by less migratory MCF-7 cells (Figure S3B) did not show an increase in metastatic potential (Figure S3D). Strikingly, when we analyzed the lungs and corresponding primary tumors of malignant MDA-MB-231 and less malignant T47D cells (Figures 7B and 7C), we observed that T47D cells that take up EVs released by the more malignant MDA-MB-231 cells showed a 52-fold increase in metastatic potential (i.e., 52-fold increase in the eGFP+ over DsRed+ ratio in lungs compared to the ratio in corresponding primary tumors) upon local communication (Figure 7B) and a 7.9-fold increase upon distant communication (Figure 7C). In addition to the less malignant T47D cells, MDA-MB-231 themselves also showed enhanced migration and metastasis upon uptake of EVs released by MDA-MB-231 cells (Figure S6), which is not surprising because mRNAs involved in metastasis were specifically loaded and enriched in EVs, and therefore, cells that take up these EVs are exposed to higher levels of mRNAs involved in metastasis. These data combined suggest that the uptake of these vesicular mRNA molecules boosts both migration and metastatic potential of tumor cells.

## Concluding Remarks

In this manuscript, we presented a technique to visualize EV transfer between tumor cells both in vitro and in living mice based on the Cre-LoxP system and study the physiological effects of this transfer. Others have tried to study the functional role of EV-mediated cell-cell communication in vivo by inhibiting multivesicular body-related EV pathways in order to render cells vesiculation deficient. However, this only led to a partial reduction in EV production (Bobrie et al., 2012; Peinado et al., 2012), probably because EV shedding from the plasma membrane was unaffected. More importantly, manipulating the EV-producing cells, by any means, may not only affect EV production, but also EV-independent factors (e.g., the cytokine profile of donor cells as observed for osteopontin, PIGF-2, PDGF-AA [Peinado et al., 2012]) with EV-independent effects on migration and metastasis of recipients cells. We took an alternative and reverse approach by using the Cre-LoxP system where the behavior of eGFP+ tumor cells (i.e., that have taken up tumor-EVs) is compared to the behavior of DsRed+ tumor cells. The observed differential behavior illustrates how uptake of EVs released from Cre+ cells affects recipient cells without the requirement to manipulate EV-releasing cells. An additional advantage of the Cre-LoxP system is that it allows detection of uptake of only the EVs released from an a priori defined cell population. Using this method, we showed directional transfer of biomolecules between tumor cells and also between tumor and non-tumor cells (Figure 5). The latter observation is in line with previous findings that bone-marrow-derived cells that take up EVs can promote metastatic niche formation (Peinado et al., 2012).

As with any technique, the Cre-LoxP system also has downsides. First, a small fraction of the Cre exchange that we detected may have occurred via EV-independent mechanisms, despite that we excluded cell-cell fusion and Cre transfer through direct cell-cell contact and despite that we observed only color switching upon intratumoral injection of purified Cre+ EVs and not upon injection of non-vesicular free Cre. Second,

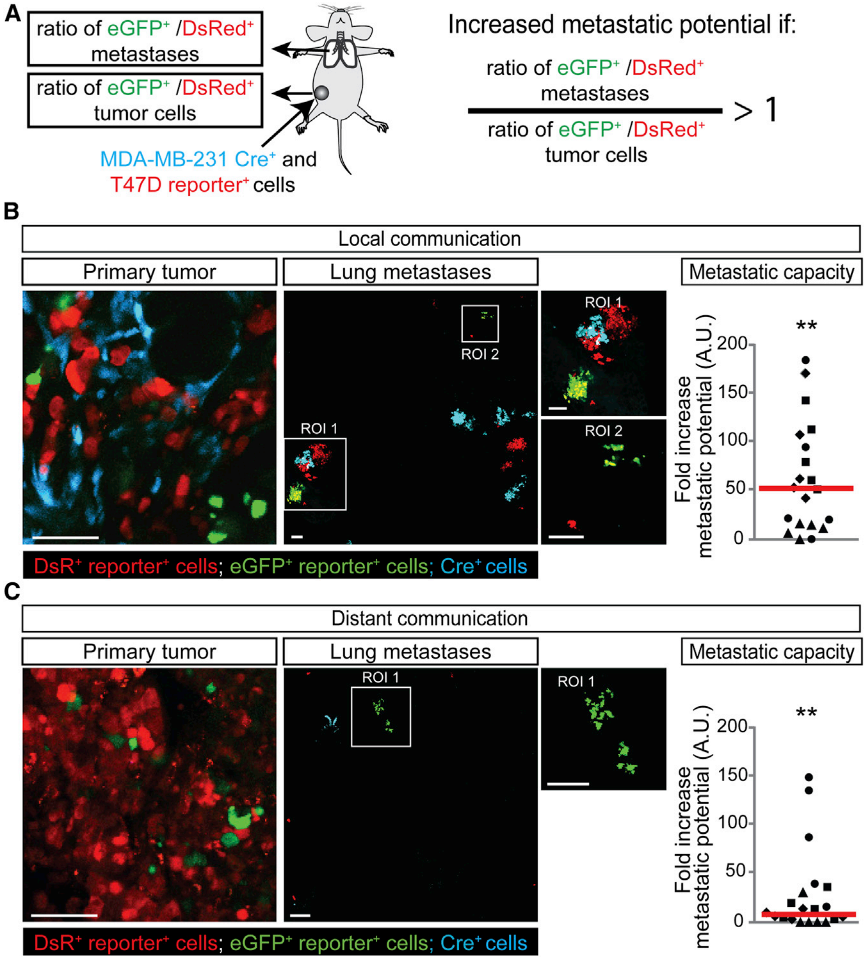


**Figure 7. Uptake of MDA-MB-231-Released EVs by T47D Cells Leads to Increased Metastatic Potential**

(A) MDA-MB-231 CFP+ Cre+ cells and T47D reporter+ cells were injected as a mixture in one mammary gland (local communication) or separately in contralateral mammary glands (distant communication). The lungs and corresponding primary reporter tumors were analyzed, and the ratio of GFP+/DsRed+ metastases and cells was determined. If EV uptake results in an enhanced metastatic potential, the ratio of eGFP+ over DsRed+ cells that arrive and grow out in the lung is expected to be higher than the ratio of these populations of cells in the primary tumor.

(B and C) Representative images of frozen sections of the primary tumor and lungs of mice of local (B) and distant communication experiments (C). Right dot plots show the fold increase of metastatic potential (increase in eGFP+ over DsRed+ ratio in lungs compared to the ratio in corresponding primary tumors). Red lines show the median, each symbol represents one cryosection, and different symbols represent different mice (n = 4 mice per condition). Scale bars represent 50 μm.

See also Figures S3 and S6.

the functional transfer of Cre does not identify the exact functional biomolecules that are transferred. Although we found a set of enriched mRNAs of genes involved in migration and metastasis, our Cre-LoxP system only reports EV uptake and does not specify transfer of any type of functional biomolecules such as DNA, (signaling) proteins, lipids, and microRNAs.

We found the mRNA of >200 genes to be vesicular enriched compared to the MDA-MB-231 cells that release these EVs. The enrichment of these biomolecules can be explained by specific loading (e.g., Villarroya-Beltri et al., 2013) or by non-specific loading of biomolecules located at peripheral cell region in EVs that are released at the edge of cells. The transfer of metastatic capacity as reported by our Cre-LoxP system is therefore likely to be caused by the transfer of multiple specifically and non-specifically loaded functional biomolecules, such as DNA, (signaling) proteins, lipids, microRNAs, and mRNAs that concertedly affect the multiple parallel migration and metastasis pathways. The molecules responsible for phenotypic switches in recipient cells cannot be generalized; different recipient cell types with expression profiles that differ from the EV-producing cells may also get affected by biomolecules that are abundantly present but not necessarily enriched in EVs. Therefore, the vesicular mRNA from the genes listed in Figure 1F affect MDA-MB-231 cells, whereas mRNA from yet another set of genes loaded in the same EVs may alter the behavior of other cell types.

Our study provides evidence in the physiological context of heterogeneous tumors inside whole organisms that tumor cells take up in-vivo-released tumor EVs. The provided proof here for the existence of EV transfer between tumor cells with a role in tumor progression is supported by in vitro studies (Al-Nedawi et al., 2008; Demory Beckler et al., 2013; Higginbotham et al., 2011; O'Brien et al., 2013; Skog et al., 2008). We demonstrate, in living mice, that malignant tumor cells, through transfer of EVs, enhance the migratory behavior and metastatic capacity of less malignant cells. These data exemplify that tumor heterogeneity is much more complex than currently anticipated, which has profound consequences for our ideas on the mechanisms of tumor progression and for designing optimal treatment strategies. EV-mediated long-range cell-cell communication could indicate that metastasized cells may influence the metastatic capacity of less malignant tumor cells in the primary tumor and that tumor cells that have acquired resistance to chemotherapy may influence the therapy resistance of other tumor cells. However, more detailed future studies are required, especially in a clinical setting, before the full magnitude and clinical implications of our observations can be revealed.

## EXPERIMENTAL PROCEDURES

### Intravital Imaging

Mice were sedated using isoflurane inhalation anesthesia (1.5% to 2% isoflurane/$O_2$ mixture). The imaging site was surgically exposed, and the mouse

was placed with its head in a facemask within a custom-designed imaging box. The isoflurane was introduced through the facemask and ventilated by an outlet on the other side of the box. The imaging box and microscope were kept at 36.5°C by a climate chamber that surrounds the whole stage of the microscope, including the objectives. Imaging was performed on an inverted Leica TCS SP5 AOBS multi-photon microscope (Mannheim, Germany) with a chameleon Ti:Sapphire pumped Optical Parametric Oscillator (Coherent Inc.). The microscope is equipped with four non-descanned detectors: NDD1 (<455 nm), NDD2 (455–490 nm), NDD3 (500–550 nm), and NDD4 (560–650 nm). Different wavelengths between 700 nm and 1,150 nm were used for excitation; CFP was excited with a wavelength of 840 nm, and GFP and DsRed were excited with a wavelength of 960 nm. CFP was detected in NND2, GFP was detected in NDD3, and DsRed was detected in NDD4. All images were in 12 bit and acquired with a 25× (HCX IRAPO N.A. 0.95 WD 2.5 mm) water objective. All pictures were processed using ImageJ software; pictures were converted to an 8 bit RGB, smoothed (if necessary), cropped (if necessary), rotated (if necessary), and contrasted linearly.

### Tracking Migration of Tumor Cells

The eGFP and DsRed images of tumors were acquired in living mice using our multi-photon microscope. Individual DsRed$^+$ and eGFP$^+$ cells can be recognized equally well, and migration differences caused by detection differences can thus be excluded. Three-dimensional volumes (z stacks) were collected, and the images were stored on the hard-disk for analysis. Images were corrected for *XYZ*-drift using custom-made software. Cells were tracked manually with an ImageJ plugin ("Manual Tracking" Rasband, W.S., ImageJ, U.S. NIH; http://imagej.nih.gov/ij/). At the beginning of each movie, a random DsRed$^+$ cell close to an eGFP$^+$ cell was selected. The *XY* position was determined over time, and the displacement and track distance were calculated by Excel (Microsoft).

### Analyzing Metastatic Capacity of Tumor Cells

Cryosections (150 μm thick) of tumors and lungs were prepared and imaged as described in the Extended Experimental Procedures section "Immunostainings and Confocal Microscopy of Tissue Sections." The images were coded randomly by one investigator, and the images were blinded analyzed by two independent researchers that did not know the experimental conditions. Tile scans of tumors were analyzed using ImageJ; a binary threshold was set separately for the red and green channels, the number of pixels above the threshold (being positive for either red or green) was measured, and the ratio eGFP/DsRed was determined. Tile scans of lungs were analyzed in LAS-AF; the amount of DsRed$^+$ and eGFP$^+$ metastases was counted manually, and the ratio eGFP/DsRed was determined. We classified metastases into two categories; metastases that were entirely DsRed$^+$ and metastases that were entirely eGFP$^+$. Only a few metastases (< 2.5%) were multicolored and therefore excluded from our analysis. Metastatic potential is calculated by dividing the ratio of eGFP$^+$ over DsRed$^+$ cells that arrive and grow out in the lung by the ratio of these populations of cells in the primary tumor.

### Gene Expression Analysis

Total RNA was isolated from cells and EVs using the TRIZOL reagent according to the manufacturer's instructions (Invitrogen) and quantified using a ND1000 spectrophotometer (Nanodrop Technologies). The quality control, RNA labeling, hybridization, and data extraction were performed at ServiceXS B.V. The RNA quality and integrity were determined using Lab-on-Chip analysis on the Agilent 2100 Bioanalyzer (Agilent Technologies, Inc.). Biotinylated cRNA was prepared using the Illumina TotalPrep RNA Amplification Kit (Ambion, Inc.) according to the manufacturer's specifications with an input of 200 ng total RNA. Per sample, 750 ng of the obtained biotinylated cRNA samples was hybridized onto the Illumina HumanHT-12 v4 (Illumina, Inc.). Each BeadChip contains 12 arrays. Hybridization and washing were performed according to the Illumina Manual "Direct Hybridization Assay Guide." Scanning was performed on the Illumina iScan (Illumina, Inc.). Image analysis and extraction of raw expression data were performed with Illumina GenomeStudio v2011.1 Gene Expression software with default settings (no background subtraction and no normalization). Primary gene expression analysis of the scanned BeadChip arrays was performed using Illumina's GenomeStudio v.2011.1 software with the default settings advised by Illumina. The data were analyzed with the R/Bioconductor package limma (Wang et al., 2013). To remove non-linear hybridization artifacts, we first performed loess normalization. Next, we combined replicates to calculate p values for differential expression. Finally, we took a 2-fold cut-off to select only the most strongly enriched RNAs in the vesicles compared to whole-cell RNA. The set of enriched genes was analyzed using the WebGestalt tool for functional classification. The set of enriched RNAs was used as the input set; the genes present on the array were used as a background set. The data discussed in this publication have been deposited in NCBI's Gene Expression Omnibus (Edgar et al., 2002) and are accessible through GEO series accession number GEO: GSE66488 (http://www.ncbi.nlm.nih.gov/geo/query/acc.cgi?acc=GSE66488).

### Statistical Analyses

For all normal distributed measurements, the Student's t test was used to determine whether there was a significant difference between two means ($p < 0.05$), and for all others the Mann-Whitney U test was used. Significance is marked with one asterisk when the p value is equal to or smaller than 0.05, and with two asterisks when the p value is equal to or smaller than 0.01.

Supplemental Experimental Procedures are available in the Supplemental Information section.

## ACCESSION NUMBERS

The data discussed in this publication have been deposited in NCBI's Gene Expression Omnibus (Edgar et al., 2002) and are accessible through GEO series accession number GEO: GSE66488.

## SUPPLEMENTAL INFORMATION

Supplemental Information includes Supplemental Experimental Procedures, six figures, two tables, and five movies and can be found with this article online at http://dx.doi.org/10.1016/j.cell.2015.04.042.

## AUTHOR CONTRIBUTIONS

J.v.R., with the help of T.W. and D.M.P., conceived the study. A.Z., J.v.R., T.W., and D.M.P. designed the experiments, and A.Z. performed most of the experiments. C.M. performed the transwell, co-culture experiments and performed immunostainings, confocal imaging, and imaging analysis. F.J.V. performed the Western blot experiments; A.K. and R.S. isolated EVs, isolated RNA, and performed RT-PCR experiments; E.B. performed confocal imaging of tissue sections; and R.M.S. performed electron microscopy and the NanoSight particle analysis. J.B., T.W., and E.d.W. performed the gene expression array analysis. J.v.R., A.Z., S.I.J.E., D.M.P., and T.W. wrote the manuscript, which was reviewed by all authors.

## ACKNOWLEDGMENTS

We thank all members of the van Rheenen group for critically reading this manuscript, and we thank Anko de Graaff and the Hubrecht Imaging Center for imaging support. We thank Wouter Karthaus for his help with cloning the floxed-DsRed-floxed-eGFP construct, Chloé Prunier for imaging assistance, and Marcel Fens for assistance at the nanosight. This work was supported by the VENI fellowships 91696087 (M.P.) and 700.10.402 (E.d.W.); VIDI fellowships 91710330 (J.v.R.), 91711366 (T.W.), and a research grant 823.02.017 (J.v.R. from the Dutch Organization of Scientific Research (NWO); grants from the Dutch Cancer Society (KWF): HUBR 2009-4621 (A.Z. and J.v.R.) and VU 2009-3775 (M.P. and F.V.); European Research Council Starting Researchers grants 260627 (R.S.) and 336540 (T.W.); Association for International Cancer Research (AICR; 13-0297; J.v.R.); and equipment grants (175.010.2007.00 and 834.11.002) from the Dutch Organization of Scientific Research (NWO).

Received: June 4, 2014
Revised: February 6, 2015
Accepted: April 2, 2015
Published: May 21, 2015

## REFERENCES

Al-Nedawi, K., Meehan, B., Micallef, J., Lhotak, V., May, L., Guha, A., and Rak, J. (2008). Intercellular transfer of the oncogenic receptor EGFRvIII by microvesicles derived from tumour cells. Nat. Cell Biol. *10*, 619–624.

Balaj, L., Lessard, R., Dai, L., Cho, Y.J., Pomeroy, S.L., Breakefield, X.O., and Skog, J. (2011). Tumour microvesicles contain retrotransposon elements and amplified oncogene sequences. Nat. Commun. *2*, 180.

Bissell, M.J., and Hines, W.C. (2011). Why don't we get more cancer? A proposed role of the microenvironment in restraining cancer progression. Nat. Med. *17*, 320–329.

Bobrie, A., and Théry, C. (2013). Exosomes and communication between tumours and the immune system: are all exosomes equal? Biochem. Soc. Trans. *41*, 263–267.

Bobrie, A., Krumeich, S., Reyal, F., Recchi, C., Moita, L.F., Seabra, M.C., Ostrowski, M., and Théry, C. (2012). Rab27a supports exosome-dependent and -independent mechanisms that modify the tumor microenvironment and can promote tumor progression. Cancer Res. *72*, 4920–4930.

Cai, Z., Yang, F., Yu, L., Yu, Z., Jiang, L., Wang, Q., Yang, Y., Wang, L., Cao, X., and Wang, J. (2012). Activated T cell exosomes promote tumor invasion via Fas signaling pathway. J. Immunol. *188*, 5954–5961.

Condeelis, J., and Segall, J.E. (2003). Intravital imaging of cell movement in tumours. Nat. Rev. Cancer *3*, 921–930.

D'Souza-Schorey, C., and Clancy, J.W. (2012). Tumor-derived microvesicles: shedding light on novel microenvironment modulators and prospective cancer biomarkers. Genes Dev. *26*, 1287–1299.

Demory Beckler, M., Higginbotham, J.N., Franklin, J.L., Ham, A.J., Halvey, P.J., Imasuen, I.E., Whitwell, C., Li, M., Liebler, D.C., and Coffey, R.J. (2013). Proteomic analysis of exosomes from mutant KRAS colon cancer cells identifies intercellular transfer of mutant KRAS. Mol. Cell. Proteomics *12*, 343–355.

Edgar, R., Domrachev, M., and Lash, A.E. (2002). Gene Expression Omnibus: NCBI gene expression and hybridization array data repository. Nucleic Acids Res. *30*, 207–210.

Ellenbroek, S.I., and van Rheenen, J. (2014). Imaging hallmarks of cancer in living mice. Nat. Rev. Cancer *14*, 406–418.

Hanahan, D., and Weinberg, R.A. (2000). The hallmarks of cancer. Cell *100*, 57–70.

Hanahan, D., and Weinberg, R.A. (2011). Hallmarks of cancer: the next generation. Cell *144*, 646–674.

Higginbotham, J.N., Demory Beckler, M., Gephart, J.D., Franklin, J.L., Bogatcheva, G., Kremers, G.J., Piston, D.W., Ayers, G.D., McConnell, R.E., Tyska, M.J., and Coffey, R.J. (2011). Amphiregulin exosomes increase cancer cell invasion. Curr. Biol. *21*, 779–786.

Hood, J.L., San, R.S., and Wickline, S.A. (2011). Exosomes released by melanoma cells prepare sentinel lymph nodes for tumor metastasis. Cancer Res. *71*, 3792–3801.

Joyce, J.A., and Pollard, J.W. (2009). Microenvironmental regulation of metastasis. Nat. Rev. Cancer *9*, 239–252.

Luga, V., Zhang, L., Viloria-Petit, A.M., Ogunjimi, A.A., Inanlou, M.R., Chiu, E., Buchanan, M., Hosein, A.N., Basik, M., and Wrana, J.L. (2012). Exosomes mediate stromal mobilization of autocrine Wnt-PCP signaling in breast cancer cell migration. Cell *151*, 1542–1556.

Meacham, C.E., and Morrison, S.J. (2013). Tumour heterogeneity and cancer cell plasticity. Nature *501*, 328–337.

Nguyen, D.X., Bos, P.D., and Massagué, J. (2009). Metastasis: from dissemination to organ-specific colonization. Nat. Rev. Cancer *9*, 274–284.

O'Brien, K., Rani, S., Corcoran, C., Wallace, R., Hughes, L., Friel, A.M., McDonnell, S., Crown, J., Radomski, M.W., and O'Driscoll, L. (2013). Exosomes from triple-negative breast cancer cells can transfer phenotypic traits representing their cells of origin to secondary cells. Eur. J. Cancer *49*, 1845–1859.

Peinado, H., Alečković, M., Lavotshkin, S., Matei, I., Costa-Silva, B., Moreno-Bueno, G., Hergueta-Redondo, M., Williams, C., García-Santos, G., Ghajar, C., et al. (2012). Melanoma exosomes educate bone marrow progenitor cells toward a pro-metastatic phenotype through MET. Nat. Med. *18*, 883–891.

Postovit, L.M., Seftor, E.A., Seftor, R.E., and Hendrix, M.J. (2006). A three-dimensional model to study the epigenetic effects induced by the microenvironment of human embryonic stem cells. Stem Cells *24*, 501–505.

Raposo, G., and Stoorvogel, W. (2013). Extracellular vesicles: exosomes, microvesicles, and friends. J. Cell Biol. *200*, 373–383.

Ratajczak, J., Miekus, K., Kucia, M., Zhang, J., Reca, R., Dvorak, P., and Ratajczak, M.Z. (2006). Embryonic stem cell-derived microvesicles reprogram hematopoietic progenitors: evidence for horizontal transfer of mRNA and protein delivery. Leukemia *20*, 847–856.

Salomon, C., Ryan, J., Sobrevia, L., Kobayashi, M., Ashman, K., Mitchell, M., and Rice, G.E. (2013). Exosomal signaling during hypoxia mediates microvascular endothelial cell migration and vasculogenesis. PLoS ONE *8*, e68451.

Skog, J., Würdinger, T., van Rijn, S., Meijer, D.H., Gainche, L., Sena-Esteves, M., Curry, W.T., Jr., Carter, B.S., Krichevsky, A.M., and Breakefield, X.O. (2008). Glioblastoma microvesicles transport RNA and proteins that promote tumour growth and provide diagnostic biomarkers. Nat. Cell Biol. *10*, 1470–1476.

Théry, C. (2011). Exosomes: secreted vesicles and intercellular communications. F1000 Biol. Rep. *3*, 15.

Théry, C., Ostrowski, M., and Segura, E. (2009). Membrane vesicles as conveyors of immune responses. Nat. Rev. Immunol. *9*, 581–593.

Valadi, H., Ekström, K., Bossios, A., Sjöstrand, M., Lee, J.J., and Lötvall, J.O. (2007). Exosome-mediated transfer of mRNAs and microRNAs is a novel mechanism of genetic exchange between cells. Nat. Cell Biol. *9*, 654–659.

Villarroya-Beltri, C., Gutiérrez-Vázquez, C., Sánchez-Cabo, F., Pérez-Hernández, D., Vázquez, J., Martin-Cofreces, N., Martinez-Herrera, D.J., Pascual-Montano, A., Mittelbrunn, M., and Sánchez-Madrid, F. (2013). Sumoylated hnRNPA2B1 controls the sorting of miRNAs into exosomes through binding to specific motifs. Nat. Commun. *4*, 2980.

Wang, J., Duncan, D., Shi, Z., and Zhang, B. (2013). WEB-based GEne SeT AnaLysis Toolkit (WebGestalt): update 2013. Nucleic Acids Res. *41*, W77–W83.

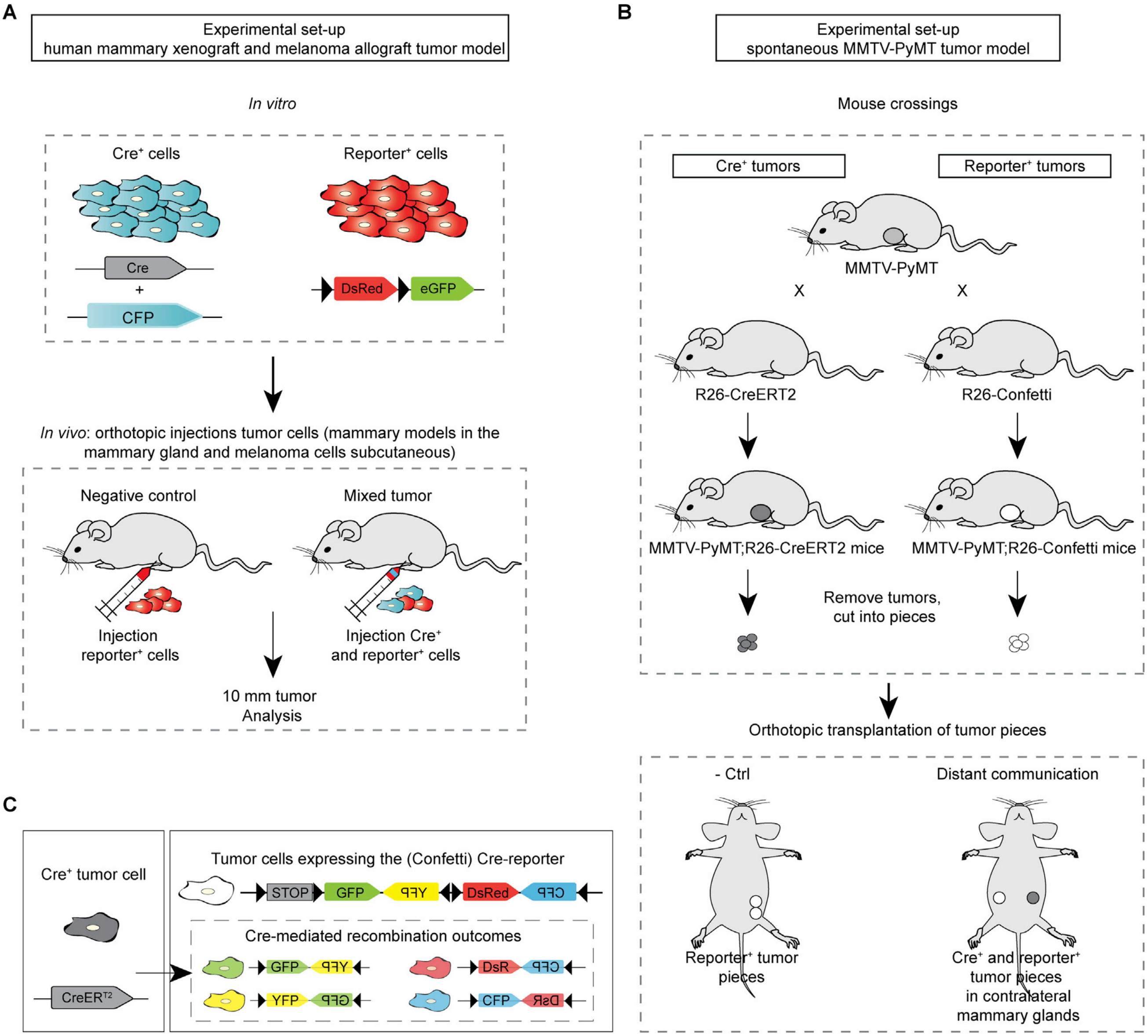


**Figure S1. The Different Mouse Models Used to Study In Vivo Vesicle Transfer, Related to Figure 4**

(A) Cartoon of the experimental setup of the human mammary xenograft and melanoma allograft model. MDA-MB-231 Cre+ and reporter+ cells were injected orthotopically (in the fourth mammary gland of immune-deficient mice for the human mammary tumor cells and subcutaneously in B6 mice for the melanoma model). When tumors had a diameter of 10 mm, the tumors were analyzed.

(B) Cartoon of the experimental setup of PyMT tumor model. Mice in which PyMT is driven by a mammary epithelial-specific promoter were crossed with mice expressing either $CreER^{T2}$ or the confetti reporter construct to create tumor pieces of Cre+ and Confetti-reporter+ cells respectively. Small tumor pieces were transplanted into immune-deficient mice, which grew out to large tumors within 2-4 months. Tumors were isolated once they were 10 mm and examined for the presence of fluorescent cells.

(C) Cartoon of the Cre+ and reporter+ confetti cells. Upon Cre activity, confetti-reporter+ cells express CFP, GFP, YFP or DsRed, which are all shown in green in the images of Figure 4E.

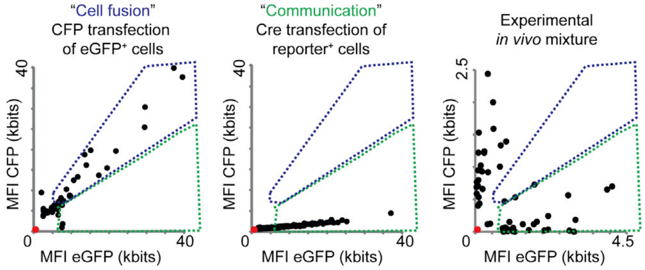


**Figure S2. In Vivo Transfer of Cre Activity Is Not Mediated by Cell-Cell Fusion, Related to Figure 4**

For 50 cells, the mean fluorescent intensity (MFI) of eGFP (reporter$^+$ cells that received Cre activity) was plotted against the MFI of CFP (Cre$^+$ cells). Intensities of non-fluorescent cells were zero (red dot). In the left plot, cell fusion was experimentally simulated by transfecting eGFP$^+$ cells with CFP DNA. In the middle plot, communication was experimentally simulated by transfecting reporter cells with Cre DNA. The right plot shows the results of the experimental in vivo local mixture of MDA-MB-231 Cre$^+$ and reporter$^+$ cells whereby the MFI of CFP and eGFP was measured for individual and randomly chosen CFP$^+$ and eGFP$^+$ cells (n = 50 cells in 3 mice). The blue dotted line outlines the cells that show cell fusion and the green dotted line shows cell communication.

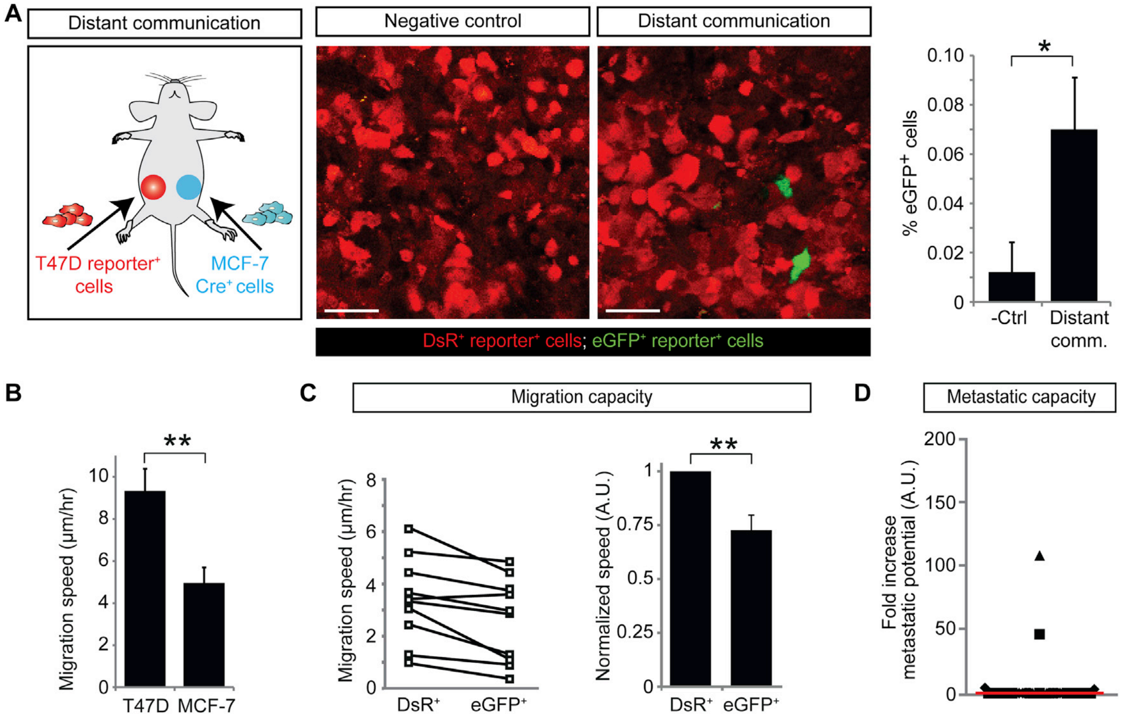


**Figure S3. T47D Cells that Take Up Less Malignant MCF-7 EVs Display a Decrease in Migration, Related to Figure 6**

(A) Left: cartoon of the experimental setup: MCF-7 CFP⁺ Cre⁺ cells and T47D reporter⁺ cells were injected separately in contralateral mammary glands. Middle: confocal images (merge of the blue, green and red channel) of the tumors in mice as indicated. Scale bar represents 50 μm. Right: quantification of the % eGFP⁺ cells in the negative control and distant communication experiment (n = 45 frozen tumor sections from 3 mice). Data are represented as mean ± SEM.

(B) The in vitro migration speed of T47D and MCF-7 cells. The cell migration was tracked over 15 hr. Experiments include at least 150 cells in five different wells in three independent experiments. Data are represented as mean ± SEM.

(C) T47D reporter⁺ tumors in mice described in A were intravitally imaged for three hours and the in vivo migration speed of eGFP⁺ and DsRed⁺ reporter⁺ cells was measured. Left: shown are the average values per imaging field. Values for eGFP⁺ and DsRed⁺ cells in the same imaging field are connected with a line (n = 10 imaging fields in 3 mice). Right: the normalized in vivo migration speed of T47D eGFP⁺ and DsRed⁺ reporter⁺ cells. Per imaging field, the values are normalized to migration speed of DsRed⁺ cells (n = 10 imaging fields in 3 mice). Data are represented as mean ± SEM.

(D) Dot plots shows the fold increase in metastatic potential by plotting the increase in the eGFP⁺ over DsRed⁺ ratio in lungs compared to the ratio in corresponding primary tumors. The red line shows the median, each symbol represents one cryosection, and the different symbols represent different mice (n = 7 mice) showing the variation within one mouse and between different mice.

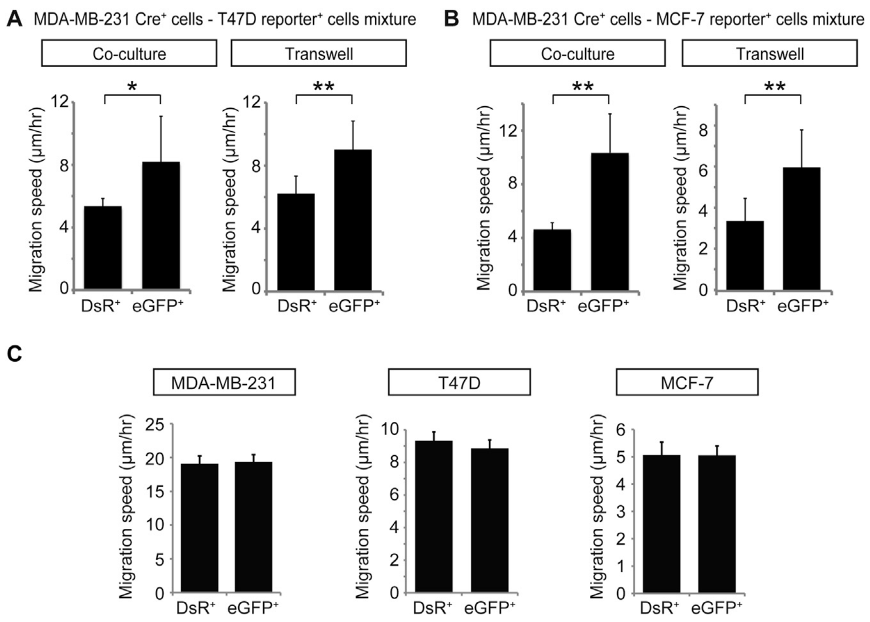


**Figure S4. EV Exchange Leads to Enhanced Migration in Recipient T47D and MCF-7 Cells, Related to Figure 6**

(A) Migration speed of T47D DsRed$^+$ and eGFP$^+$ reporter$^+$ cells that are cultured together with MDA-MB-231 Cre$^+$ cells in a 1:1 ratio (co-culture; n = 3 independent experiments) or that are cultured with MDA-MB-231 Cre$^+$ cells seeded on top of a 400 nm transwell membrane (transwell; n = 3 independent experiments). Data are represented as mean ± SEM.

(B) Migration speed of MCF-7 DsRed$^+$ and eGFP$^+$ reporter$^+$ cells that are cultured together with MDA-MB-231 Cre$^+$ cells in a 1:1 ratio (co-culture; n = 3 independent experiments) or that are cultured with MDA-MB-231 Cre$^+$ cells seeded on top of a 400 nm transwell membrane (transwell; n = 3 independent experiments). Data are represented as mean ± SEM.

(C) MDA-MB-231, T47D and MCF-7 reporter$^+$ cells were transiently transfected with Cre. The in vitro migration of DsRed$^+$ and eGFP$^+$ cells was tracked over 15 hr. Experiments include at least 150 cells per condition in five different wells in three independent experiments. Data are represented as mean ± SEM.

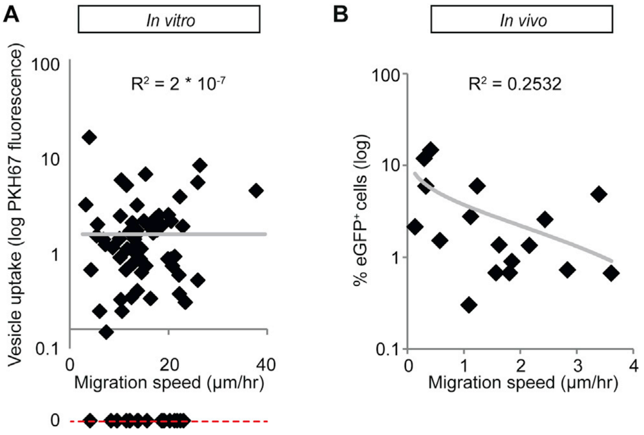


**Figure S5. Migratory Cells Do Not Have A Priori Higher Capacity to Take Up EVs, Related to Figure 6**

(A) The in vitro migration of MDA-MB-231 cells was tracked over 6 hr. After the migration experiment, PKH67-labeled MDA-MB-231-derived EVs were added and the uptake was monitored. The migration speed of the cell was measured and plotted against the vesicle uptake, expressed as the total PKH67 fluorescence per cell (n = 78 cells; trend line shown in gray). The cells that did not take up any PKH67 could not be shown in the graph and are therefore indicated at the red dashed line. Note that the $R^2$ is close to zero indicating that there is no correlation between migration speed and EV uptake.

(B) The average in vivo migration speed per imaging field and the percentage of T47D eGFP$^+$ reporter cells in tumors that report Cre activity from distant MDA-MB-231 Cre$^+$ cells transplanted in contralateral glands (Figure 6A) (n = 17 imaging fields in 3 mice; trend line shown in gray).

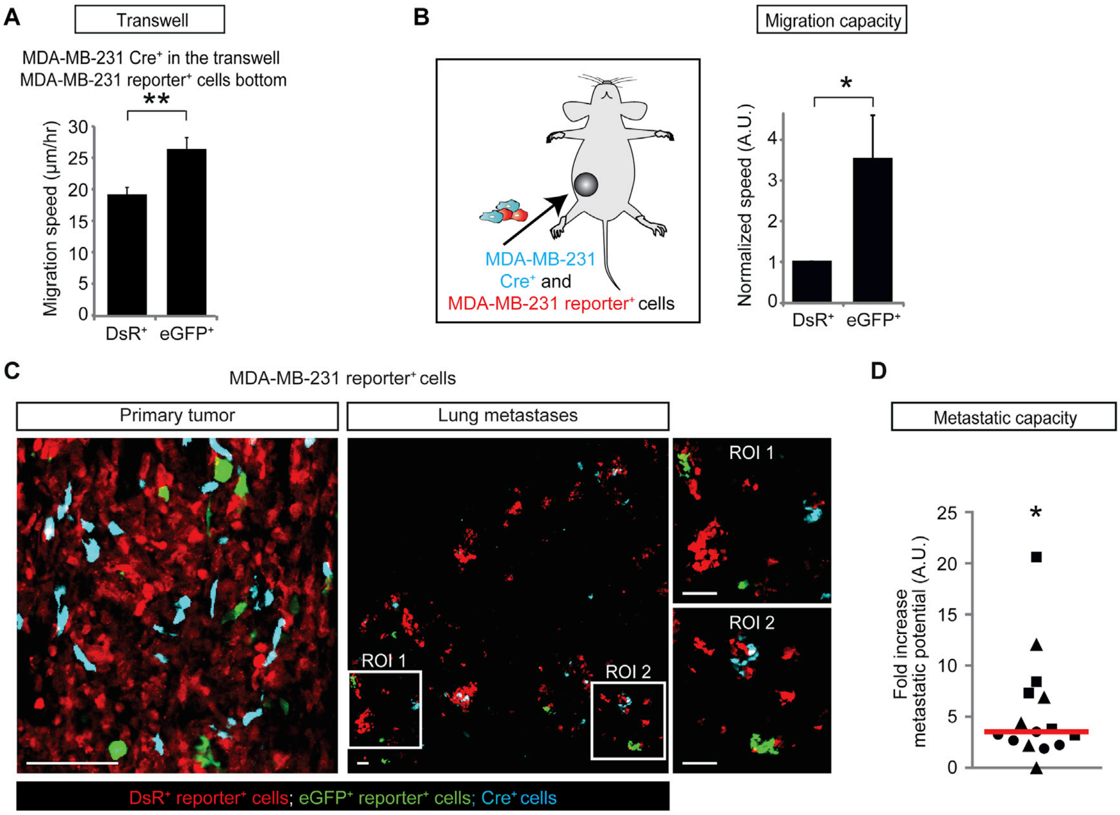


**Figure S6. Biomolecule Exchange between MDA-MB-231 Cells Leads to Enhanced Metastasis Formation, Related to Figure 7**

(A) Graph shows the migration speed of MDA-MB-231 DsRed+ and eGFP+ reporter+ cells that are cultured with MDA-MB-231 Cre+ cells seeded on top of a 400 nm transwell membrane (n = 3 independent experiments). Data are represented as mean ± SEM.

(B) Left: cartoon of the experimental setup, where a mixture of MDA-MB-231 CFP+ Cre+ cells and reporter cells were injected in the mammary gland and the subsequent tumors were intravitally imaged for three hours. Right: the normalized in vivo migration speed of MDA-MB-231 eGFP+ and DsRed+ reporter+ cells. Per imaging field, the values are normalized to migration speed of DsRed+ cells (n = 15 imaging fields in 3 mice). Data are represented as mean ± SEM.

(C) Shown are representative images of frozen sections of the primary tumor and lungs of mice injected with a mixture of MDA-MB-231 CFP+ Cre+ and reporter+ cells (n = 3 mice). The images of the lung are maximum projection images of a volume of 150 μm. Scale bars represent 100 μm.

(D) Dot plot shows the fold increase of metastatic potential by plotting the increase in the eGFP+ over DsRed+ ratio in lungs compared to the ratio in corresponding primary tumors. The red line shows the median, each symbol represents one cryosection, and the different symbols represent different mice (n = 3 mice) showing the variation within one mouse and between different mice. The *P*-values were calculated using Mann-Whitney U-test using the mouse as comparative unit.
